# TLR4 signaling improves PD-1 blockade therapy during chronic viral infection

**DOI:** 10.1371/journal.ppat.1007583

**Published:** 2019-02-06

**Authors:** Yidan Wang, Young Rock Chung, Simon Eitzinger, Nicole Palacio, Shana Gregory, Mitra Bhattacharyya, Pablo Penaloza-MacMaster

**Affiliations:** Department of Microbiology-Immunology, Feinberg School of Medicine, Northwestern University, Chicago, IL, United States of America; Nationwide Children's Hospital, UNITED STATES

## Abstract

CD8 T cells are necessary for the elimination of intracellular pathogens, but during chronic viral infections, CD8 T cells become exhausted and unable to control the persistent infection. Programmed cell death-1 (PD-1) blockade therapies have been shown to improve CD8 T cell responses during chronic viral infections. These therapies have been licensed to treat cancers in humans, but they have not yet been licensed to treat chronic viral infections because limited benefit is seen in pre-clinical animal models of chronic infection. In the present study, we investigated whether TLR4 triggering could improve PD-1 therapy during a chronic viral infection. Using the model of chronic lymphocytic choriomeningitis virus (LCMV) infection in mice, we show that TLR4 triggering with sublethal doses of lipopolysaccharide (LPS) followed by PD-1 blockade results in superior improvement in circulating virus-specific CD8 T cell responses, relative to PD-1 blockade alone. Moreover, we show that the synergy between LPS and PD-1 blockade is dependent on B7 costimulation and mediated by a dendritic cell (DC) intrinsic mechanism. Systemic LPS administration may have safety concerns, motivating us to devise a safer regimen. We show that *ex vivo* activation of DCs with LPS, followed by adoptive DC transfer, results in a similar potentiation of PD-1 therapy without inducing wasting disease. In summary, our data demonstrate a previously unidentified role for LPS/TLR4 signaling in modulating the host response to PD-1 therapy. These findings may be important for developing novel checkpoint therapies against chronic viral infection.

## Author summary

Although PD-1 therapies have revolutionized cancer treatment, these therapies have not yet been licensed to treat chronic viral infections. This is because limited benefit is seen in pre-clinical models of chronic viral infection. Interestingly, recent reports in cancer models have suggested that certain microbes can affect the efficacy of PD-1 therapies, but the specific microbial products that modulate host responses to therapy remain unknown. We utilized a model of chronic viral infection to evaluate if bacterial lipopolysaccharide (LPS), a major constituent of the microbiome, influences the efficacy of PD-1 therapy. Interestingly, we demonstrate that TLR4 triggering with low doses of LPS combined with PD-1 blockade induced a synergistic rescue of exhausted virus-specific CD8 T cell responses. Moreover, we demonstrate that adoptive transfer of LPS-activated DCs also results in similar improvement of PD-1 therapy without inducing overt immunopathology. Mechanistically, the synergy was DC-intrinsic, IFN-I independent, and B7/CD28 dependent. Taken together, our data may be important for understanding how components of the microbiome modulate the efficacy of PD-1 therapy, and may result in novel combined regimens for treating chronic viral infections.

## Introduction

CD8 T cells are critical for controlling intracellular infections, but during chronic viral infections, CD8 T cells undergo functional exhaustion. Immune checkpoint blockade therapies can restore the function and proliferative capacity of exhausted CD8 T cells. In particular, PD-1 blockade therapy is now licensed to treat human cancers. This therapy was initially found to improve exhausted CD8 T cells in the chronic lymphocytic choriomeningitis virus (LCMV) infection model in mice, and subsequently, it was demonstrated to also improve exhausted CD8 T cells in other models of chronic infection [1–6]. PD-1 therapies targeting either the receptor (PD-1) or the ligand (PD-L1) can partially improve exhausted CD8 T cells, but these therapies have clinical limits that are not fully understood. In particular, their limited efficacy in models of chronic viral infection has precluded their licensing for treating chronic viral infections, and significant efforts are aimed at improving efficacy using combined regimens [7–12]. Interestingly, it has been suggested that certain products of the microbiota can improve clinical responses to PD-1 therapy [13, 14], but the specific microbial products that underpin this positive effect remain unknown.

There is a growing interest in modulating innate immune responses to improve immune checkpoint therapies. For example, TLR9 activation can improve cancer immunotherapy [15, 16]. Another study showed that stimulation of the STING pathway, which senses cytosolic dinucleotides, can improve PD-1 therapy during cancer [17]. However, it is unknown if TLR4, a potent activator of innate immune responses, affects PD-1 therapy during chronic viral infection. We first evaluated whether lipopolysaccharide (LPS), a natural component of the Gram-negative microbiome, was able to affect the host response to PD-1 therapy during a chronic LCMV infection in mice. Treatment of chronically infected mice with LPS alone did not rescue exhausted CD8 T cells. However, combined treatment with LPS and PD-1 therapy resulted in one of the most impressive synergistic effects that we have ever observed. These results identify for the first time a specific microbial product that augments the efficacy of PD-1 immunotherapy. Systemic LPS administration has obvious safety concerns that may preclude its clinical use, but we demonstrate that adoptive transfer of *ex vivo* activated DCs also synergizes with PD-1 therapy without inducing wasting disease, suggesting the potential translatability of our findings. Overall, we demonstrate novel strategies to harness the TLR4 pathway to improve PD-1 therapy during chronic viral infection.

## Results

### Bacterial LPS improves PD-1 therapy during chronic viral infection

**Fig 1:**
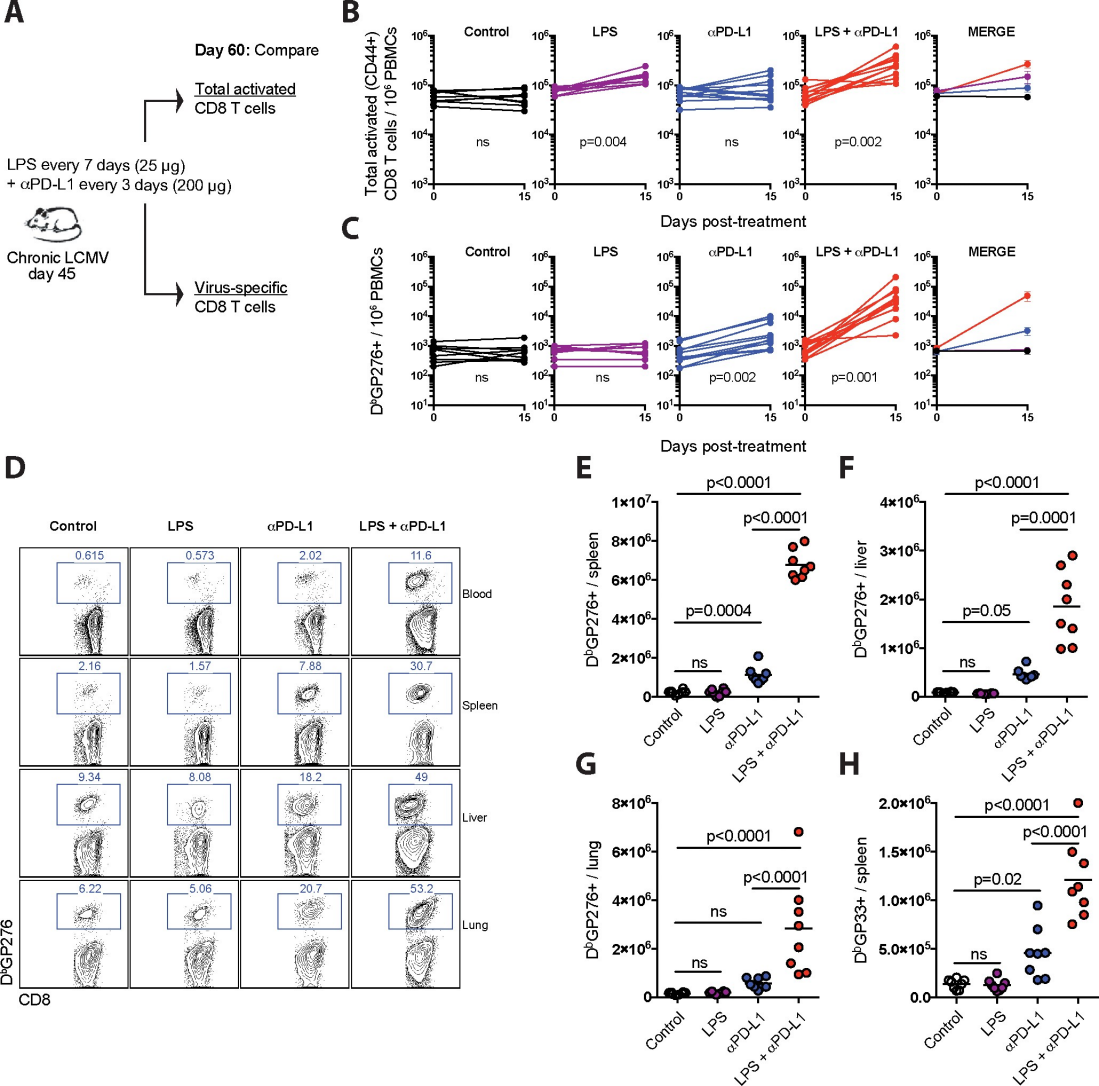
Effects of LPS on CD8 T cells in chronically infected mice. (**A**) Experimental outline for evaluating the effect of LPS on total activated CD8 T cells or virus-specific CD8 T cells during a chronic viral infection. Mice chronically infected with LCMV Cl-13 received PD-L1 blocking antibodies combined with LPS. (**B**) Summary of total activated CD8 T cells in blood. (**C**) Summary of virus-specific CD8 T cells in blood. (**D**) Representative FACS plots showing the frequencies of virus-specific (D^b^GP276+) CD8 T cells in blood and tissues. (**E**) Summary of virus-specific (D^b^GP276+) CD8 T cells in spleen. (**F**) Summary of virus-specific (D^b^GP276+) CD8 T cells in liver. (**G**) Summary of virus-specific (D^b^GP276+) CD8 T cells in lung. (**H**) Summary of virus-specific (D^b^GP33+) CD8 T cells in spleen. Data are pooled from different experiments; PBMC data are from experiments that were performed 3 times, n = 3–5 mice per experiment; Tissue data are from experiments that were performed 2 times, n = 3–5 mice per experiment; ns, not significant. Indicated p-values for panels B-C compare pre- and post-treatment values for each group using Wilcoxon matched-pairs signed rank test. All other data were analyzed using ANOVA for multiple comparisons with Holm-Sidak’s correction. Error bars represent SEM.

Prior studies have shown that LPS improves memory CD8 T cell responses [18], but whether LPS improves exhausted CD8 T cell responses remains unknown. To answer this simple question, we utilized the model of lifelong LCMV infection in mice (LCMV Cl-13). At day 45 post-infection, we treated mice with sublethal doses of LPS administered throughout a PD-L1 blockade therapy, and after 15 days, mice were sacrificed to analyze total activated CD8 T cells, as well as virus-specific CD8 T cells (Fig 1A). As expected, LPS alone induced a significant increase in total activated CD8 T cells (Fig 1B), consistent with its known adjuvant effect, but it did not increase virus-specific CD8 T cells (Fig 1C). These data demonstrate that LPS alone does not exert adjuvant effects on exhausted virus-specific CD8 T cells that sense persistent viral antigen.

**Fig 2:**
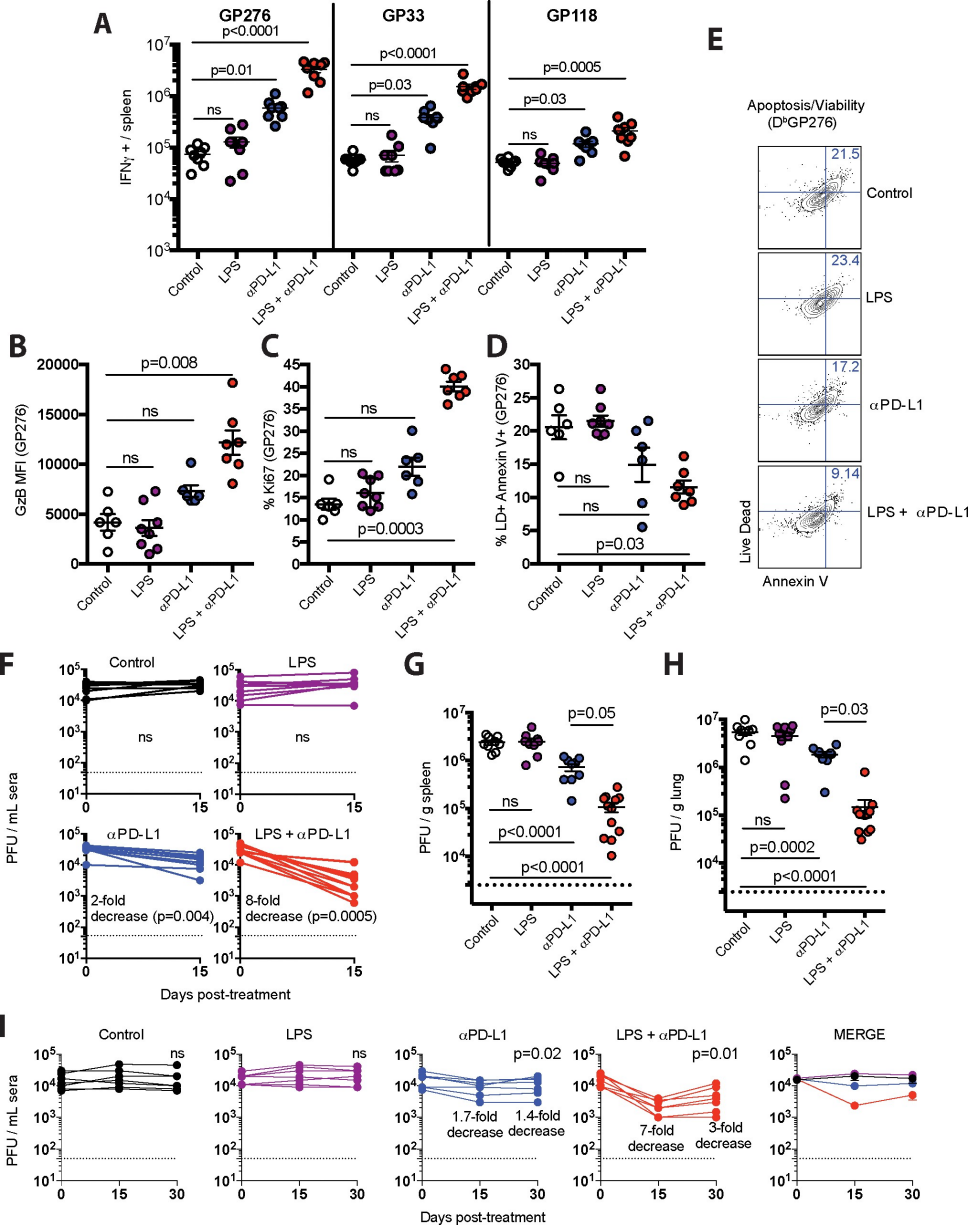
Potent synergism between LPS and PD-1 therapy. (**A**) Absolute numbers of virus-specific CD8 T cells in spleen producing IFN-γ after 5-hr stimulation with LCMV peptides (0.1 μg/mL) in the presence of brefeldin A and monensin at 37°C in 5% CO_2._ B) Granzyme B expression in virus-specific (D^b^GP276+) CD8 T cells from spleen. (**C**) Ki67 expression in virus-specific CD8 T cells from spleen. (**D**) Apoptotic (Annexin+ Live/Dead+) virus-specific CD8 T cells in spleen. (**E**) Representative FACS plots depicting apoptotic virus-specific CD8 T cells in spleen. (**F**) Summary of viral control in sera. Fold-change is calculated by dividing the pre-treatment levels by the post-treatment levels. (**G**) Summary of viral control in spleen (day 15 post-treatment). (**H**) Summary of viral control in lung (day 15 post-treatment). (**I**) Summary of long-term viral control in sera. Virologic control is maintained long-term, but complete viral elimination is not observed. Experimental layout was similar as the one depicted in Fig 1A. For all plaque assays the limit of detection is indicated by a dashed line. Data are pooled from different experiments. Experiments were performed twice, n = 3–5 mice per experiment, except for panel F that included 3 experiments, n = 3–5 mice per experiment; ns, not significant. Statistical analyses for panel A were performed with Kruskal-Wallis test with Dunn’s multiple comparison test; for panels B-D and G-H ANOVA for multiple comparisons with Holm-Sidak’s correction was used; for panels F and I, Wilcoxon matched-pairs signed rank test was used comparing pre-treatment viremia versus day 30 viremia within the same treatment group. Error bars represent SEM.

**Fig 3:**
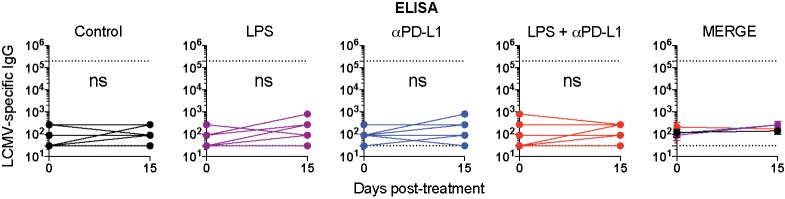
Summary of virus-specific antibody responses in sera. ELISA data are plotted as endpoint titer. Lower dashed line represents the limit of detection; upper dashed line represents the antibody titer in LCMV Armstrong immune mice that cleared infection and exhibited functional immune memory (~day 60). Data are pooled from different experiments. Experiments were performed 2 times, n = 3 mice per experiment; ns, not significant. Statistical analyses were performed using Wilcoxon matched-pairs signed rank test. Error bars represent SEM.

However, LPS rendered exhausted virus-specific CD8 T cells more responsive to PD-1 therapy. Combining LPS with PD-L1 blockade resulted in synergistic expansion of D^b^GP276+ virus-specific CD8 T cells in blood (Fig 1C). This unprecedented synergy was also evident in tissues (Fig 1D–1G), and was also observed for other responses, such as D^b^GP33-41 (Fig 1H). Virus-specific CD8 T cells also exhibited more significant functional improvement (Fig 2A), enhanced expression of granzyme B (Fig 2B) and the proliferation marker Ki67 (Fig 2C), and showed increased survival following combined therapy (Fig 2D and 2E). In addition, the combined therapy resulted in improved antiviral control in sera and tissues relative to PD-L1 blockade alone (Fig 2F–2H). We also evaluated long-term viremia in separate experiments and we observed improved virologic control for several weeks (Fig 2I). Although there were profound effects on virus-specific CD8 T cell responses, virus-specific antibody responses were not affected by the therapy (Fig 3). Taken together, these results demonstrate that bacterial LPS renders exhausted CD8 T cells more responsive to PD-L1 blockade therapy.

### Gene expression analyses of rescued CD8 T cells show differences in interferon type I and CD28 induced genes

**Fig 4:**
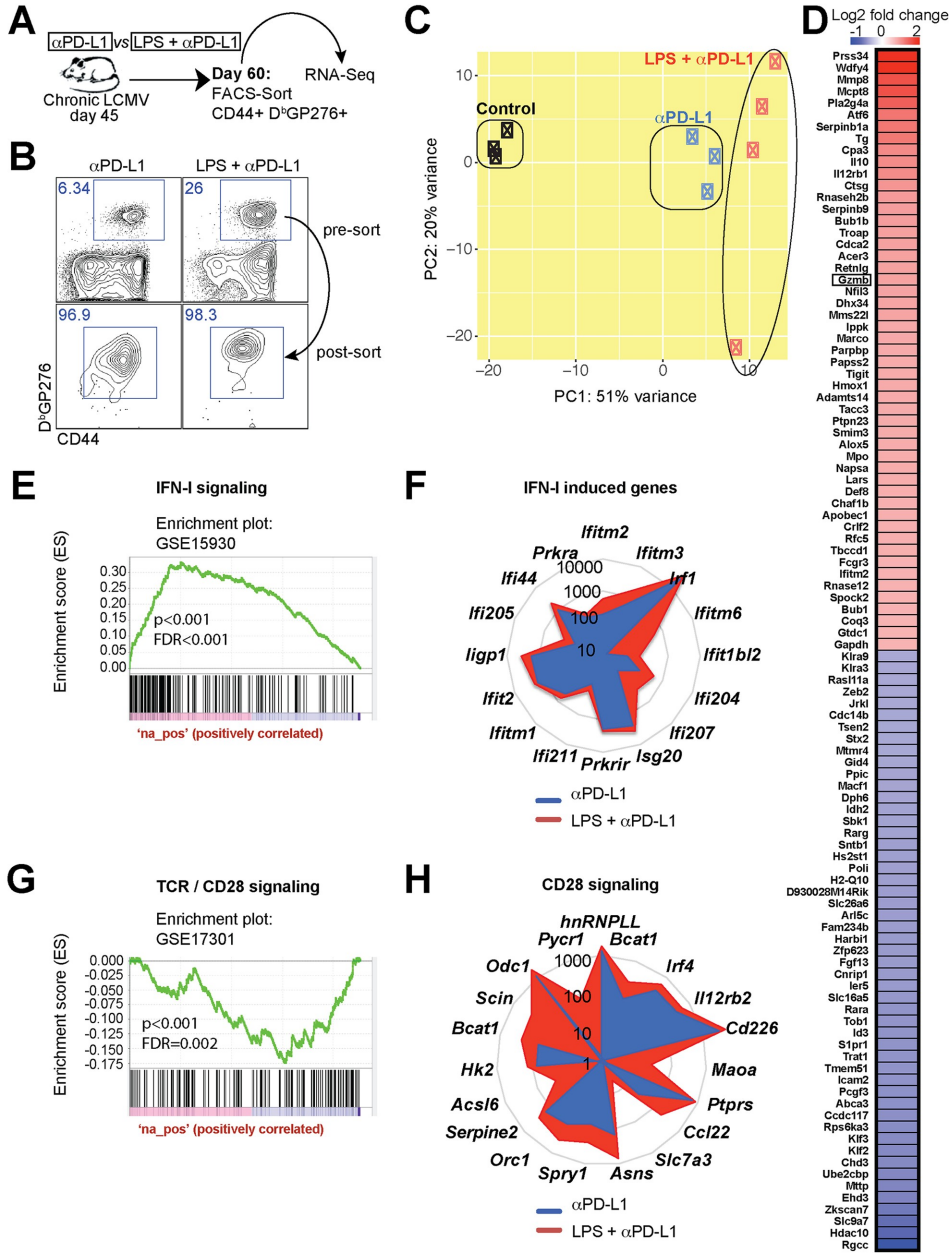
Gene expression profiling of virus-specific CD8 T cells shows enrichment in IFN-I and CD28 driven genes. (**A**) Experimental outline for comparing the transcriptional signature of virus-specific CD8 T cells. RNA-Seq was performed on FACS-sorted D^b^GP276+ CD8 T cells from spleen. (**B**) Cell purity following FACS-sorting of virus-specific CD8 T cells. (**C**) PCA comparing the transcriptional landscape of rescued CD8 T cells following PD-L1 blockade alone or combined LPS and PD-L1 blockade. (**D**) Heat map of the most differentially expressed genes between single and combined treatment. (**E**) GSEA plots demonstrating enrichment for IFN-I signaling genes in virus-specific CD8 T cells following combined therapy. (**F**) Radar plots showing expression of various IFN-I driven genes. (**G**) GSEA plots demonstrating enrichment for CD28 costimulation genes in virus-specific CD8 T cells following combined therapy. In panels E and G, DN and UP mean downregulated or upregulated, respectively, relative to previously identified transcriptional signatures used as reference. (**H**) Radar plots showing expression of various CD28 driven genes. The genes selected were shown to be enriched following CD28 costimulation in a prior publication [71]. Presented data are from one experiment, control (n = 3), PD-L1 therapy alone (n = 3), or combined LPS and PD-L1 therapy (n = 4) at day 15 post-treatment.

To understand the mechanism of how LPS renders exhausted CD8 T cells more responsive to PD-1 therapy, we performed gene expression analyses. At day 15 post-treatment, virus-specific CD8 T cells were FACS-sorted and used for RNA-Seq analyses (Fig 4A and 4B). Principal Component Analyses (PCA) showed differential clustering, suggesting differences in gene expression following combined treatment (Fig 4C). Heat map analyses showed the granzyme B gene (*Gzmb*) as one of the top upregulated genes in the combined treatment group, consistent with our flow cytometric data from Fig 2B. Revigo analyses showed enrichment in metabolic, cell division, and immune processes, such as cell killing and defense response, in the combined treatment (S1 Fig). In particular, genes that are normally induced by interferon type I signaling (IFN-I) (Fig 4E and 4F) and CD28 costimulation (Fig 4G and 4H) were highly enriched in virus-specific CD8 T cells in the combined treatment by Gene Set Enrichment Analyses (GSEA) and radar plot analyses. At the genome-wide level, the two most differentially enriched pathways were CTLA-4 and CD28 signaling by Ingenuity Pathway Analyses (IPA) (S2 Fig) and Molecular Activity Predictor (MAP) Analyses (S3 Fig). These data indicated that CTLA-4 and CD28 signaling were primarily triggered on CD8 T cells of mice receiving combined LPS and PD-L1 blockade. It is important to highlight that the CTLA-4 and CD28 receptors on CD8 T cells are triggered by the same B7.1/B7.2 molecules expressed on antigen presenting cells (APCs).

**Fig 5:**
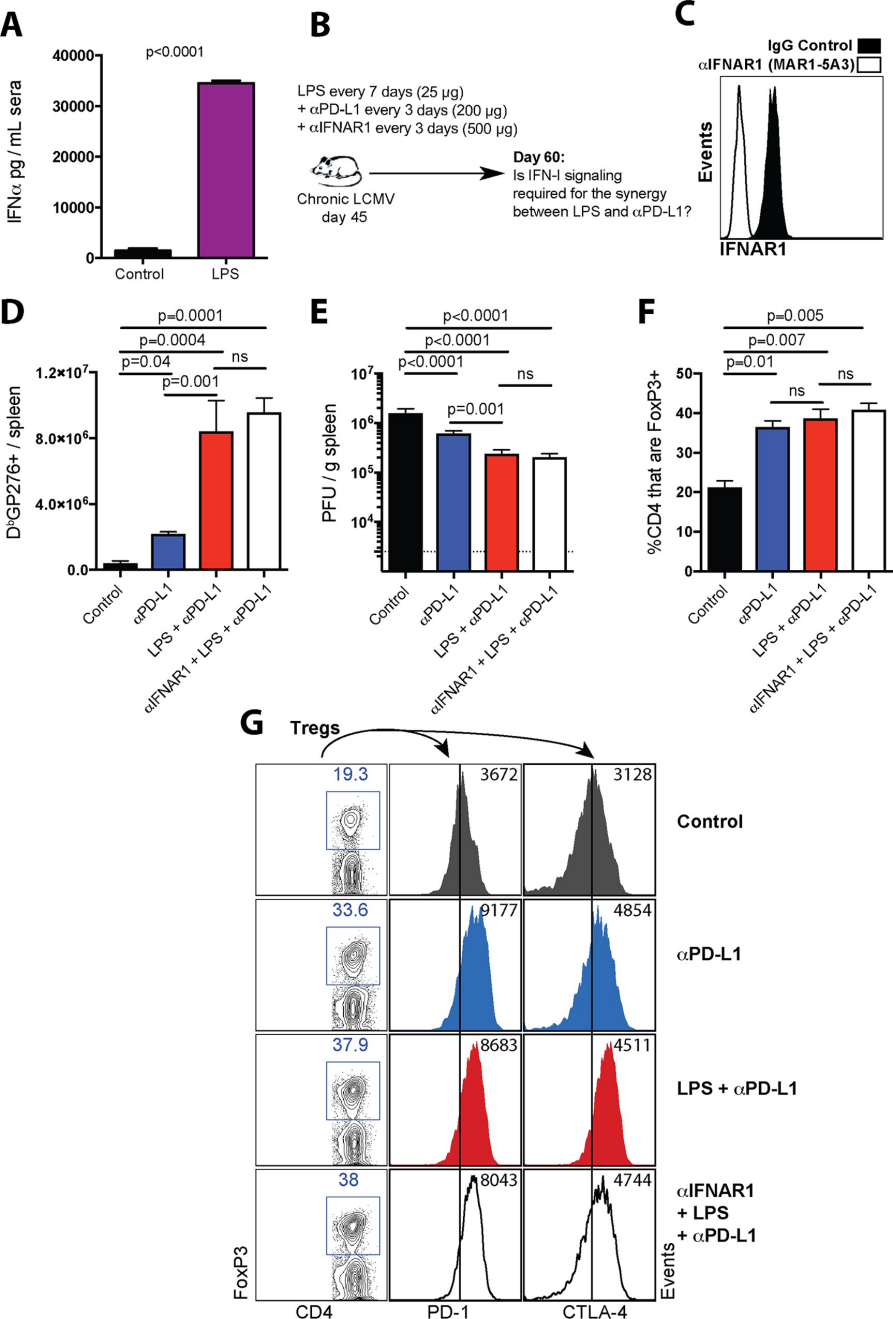
IFN- I signaling is dispensable for the potentiation of PD-1 therapy by LPS. (**A**) Systemic IFNα levels after LPS administration in chronically infected mice. Chronically infected mice (~day 45 post-infection) were treated with either PBS or LPS, and IFNα levels were quantified in sera after 24 hours. (**B**) Experimental outline for blocking interferon type I receptor. Mice chronically infected with LCMV Cl-13 received a standard PD-L1 blockade regimen combined with LPS administration and injection of IFNAR1 (MAR1-5A3) blocking antibody. (**C**) Representative FACS histogram corroborating that MAR1-5A3 antibody blocks the IFNAR1 receptor at day 3 post-treatment (gated on PBMCs). (**D**) Summary of D^b^GP276+ responses in spleen. (**E**) Summary of viral control in spleen. (**F**) Summary of Treg responses in spleen. (**G**) Representative FACS plots showing the frequencies of splenic CD4 T cells that are FoxP3+ (first column). In the second and third columns, FoxP3+ CD4 T cells were gated to quantify inhibitory receptor expression. Note that LPS treatment does not attenuate Treg responses. For plaque assays the limit of detection is indicated by a dashed line. Data are pooled from different experiments. Experiments were performed 2 times, n = 4–5 mice per experiment; ns, not significant. Statistical analyses were performed using ANOVA for multiple comparisons with Holm-Sidak’s correction. Error bars represent SEM.

Our transcriptional data suggested a possible a role for IFN-I signaling and B7/CD28 costimulation in promoting the synergy between LPS and PD-1 therapy. Consistent with the gene expression data, LPS treatment in chronically infected mice resulted in high levels of IFN-I in sera (Fig 5A). Immune activation following TLR4 triggering is thought to be dependent on IFN-I responses [19–21], leading us to hypothesize that IFN-I responses mediated the synergistic effect of LPS on PD-1 therapy. To interrogate a possible role for IFN-I responses, we treated chronically infected mice with an antibody that blocked IFN-I responses, administered together with LPS and PD-L1 blocking antibodies (Fig 5B). We utilized an antibody that binds to interferon α/β receptor subunit 1 (IFNAR1) and precludes its binding to interferons α/β, abrogating downstream IFN-I signaling [22–26]. This is a widely characterized antibody that has been previously shown to reduce the expression of interferon-stimulated genes (ISGs) during chronic viral infection *in vivo* [22]. We also confirmed *in vivo* blockade of the IFNAR1 receptor by this antibody clone (MAR1-5A3) (Fig 5C). Moreover, we confirmed that this antibody abrogated IFNα-driven PD-L1/MHC-I upregulation *in vitro* using tumor cell lines (S4 Fig), confirming that this antibody precludes IFN-I signaling. However, contrary to our hypothesis, IFN-I blockade with this antibody did not abrogate the synergistic effect of LPS and PD-L1 blockade, suggesting an IFN-I independent mechanism (Fig 5D and 5E).

We previously demonstrated that T regulatory cell (Treg) ablation induces a similar synergistic effect on PD-1 therapy [27]. Tregs suppress CD8 T cells via an APC-dependent mechanism; CTLA-4 molecules on Tregs bind to and remove B7 molecules on APCs, a process referred to as trans-endocytosis [27–29]. High PD-1 expression on Tregs is especially associated with increased Treg suppressive function [30]. We hypothesized that the synergistic effect of LPS on PD-1 therapy was associated with attenuated Treg responses. However, LPS did not attenuate Treg responses in terms of their frequency (Fig 5F) or expression of inhibitory receptors associated with Treg suppressive function [29, 31] (Fig 5G). Taken together, the synergy between LPS and PD-1 therapy was likely not caused by downregulation of Treg responses. It must be noted, however, that PD-L1 blockade itself increased Treg frequencies and expression of inhibitory markers on Tregs (Fig 5G).

During normal conditions, LPS is known to induce the expression of costimulatory molecules on DCs [32, 33]. During chronic infection, however, DC function is severely impaired [34–38], and it is unclear if TLR4 activation with LPS can override DC dysfunction and induce the expression of costimulatory molecules on DCs. To answer this question, we phenotyped DCs 24 hours after treatment of chronically infected mice with LPS. This treatment did not increase DC numbers in chronically infected mice (S5A Fig), but it induced upregulation of MHC molecules on DCs from spleen (S5B and S5C Fig). LPS also resulted in significant upregulation of B7.1 and B7.2 molecules (S5D and S5E Fig), suggesting that LPS improved DC costimulatory function during chronic viral infection. However, other TLR4 agonists (Neoseptin-3 and MPLA) and TLR2 agonists (LAM) did not improve costimulatory molecule expression during chronic viral infection (S5F Fig). Note that LPS, Neoseptin-3 and MPLA are TLR4 agonists, but these molecules are biologically distinct. In particular, LPS has been shown to be substantially more pro-inflammatory and induce distinct types of downstream responses [18, 39–41].

Notwithstanding the upregulation of costimulatory B7 molecules, LPS treatment also upregulated inhibitory PD-L1 molecules on DCs, suggesting a negative feedback loop (S5G and S5H Fig). A similar pattern of dual B7 and PD-L1 upregulation was observed on other APC subsets in chronically infected mice (S6 Fig). Dual upregulation of B7 and PD-L1 was also observed in DCs from naïve mice (S7 Fig). Overall, our data demonstrate that LPS has more profound phenotypic effects on DCs, relative to other TLR4 agonists.

**Fig 6:**
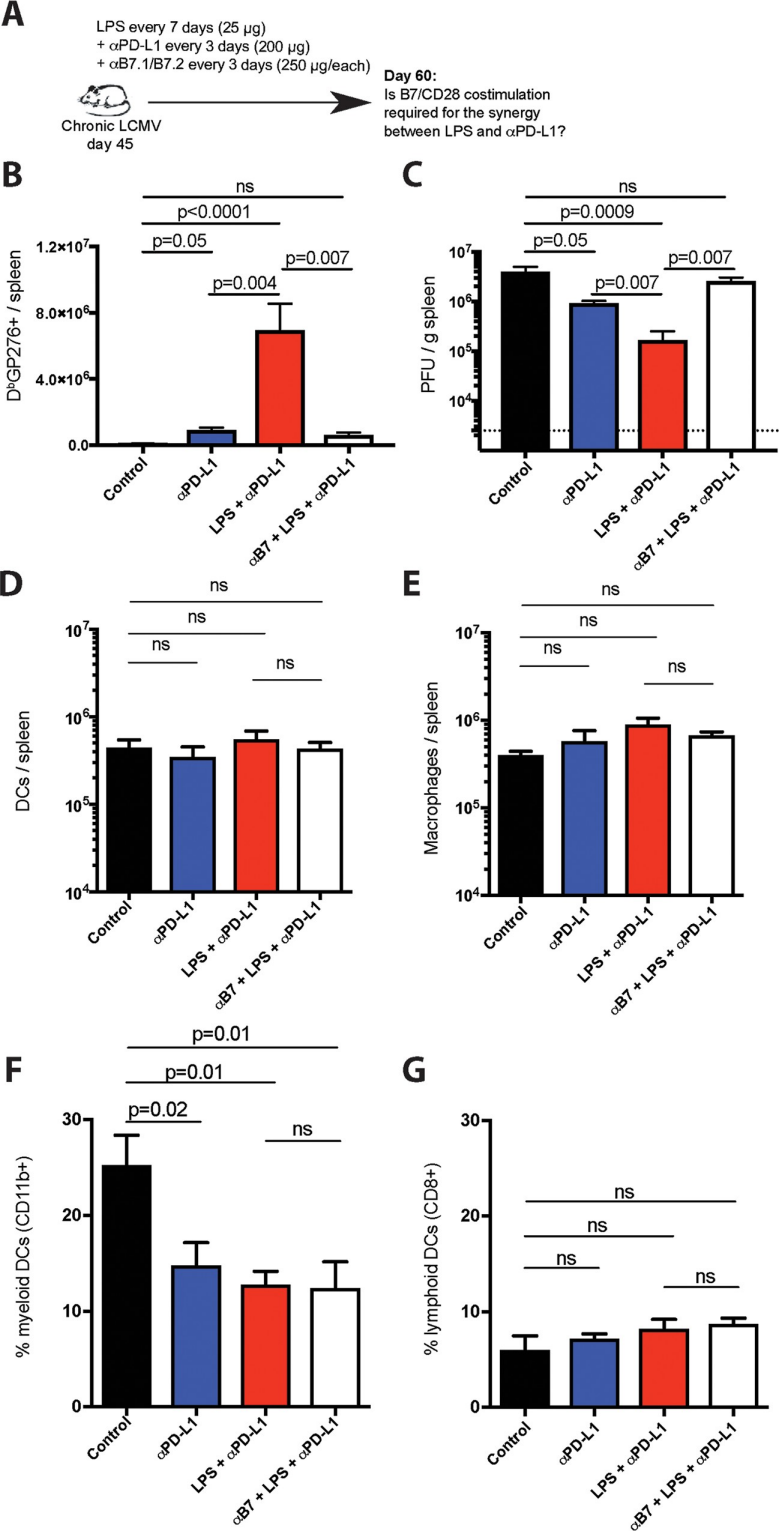
B7 costimulation is necessary for the potentiation of PD-1 therapy by LPS. (**A**) Experimental outline for blocking B7/CD28 costimulation. Mice chronically infected with LCMV Cl-13 received a standard PD-L1 blockade regimen combined with LPS administration and injection of B7.1/B7.2 blocking antibodies. (**B**) Summary of D^b^GP276+ responses in spleen. (**C**) Summary of viral control in spleen. For plaque assays the limit of detection is indicated by a dashed line. (**D**) Number of DCs in spleen. DCs were gated as live CD3- NK1.1- Ly6G- CD19- CD11c+. (**E**) Number of macrophages in spleen. Macrophages were gated as live CD3- NK1.1- CD19- F4/80+ CD11b+ (**F**) Percentage of DCs that are myeloid DCs (CD3- NK1.1- Ly6G- CD19- CD11c+ CD11b+ CD8-). (**G**) Percentage of DCs that are lymphoid DCs (CD3- NK1.1- Ly6G- CD19- CD11c+ CD11b- CD8+). Data are pooled from different experiments. Experiments were performed 2 times, n = 4–5 mice per experiment; ns, not significant. Statistical analyses were performed using ANOVA for multiple comparisons with Holm-Sidak’s correction. Error bars represent SEM.

B7 upregulation on APCs by flow cytometric analyses and enrichment in CD28-driven genes on virus-specific CD8 T cells by gene expression analyses led us to hypothesize a potential role for B7/CD28 costimulation. To test this hypothesis, we treated chronically infected mice with LPS, PD-L1 blocking antibodies, and B7.1/B7.2 blocking antibodies (Fig 6A). We utilized anti-B7 antibodies that have been previously shown to block the B7/CD28 costimulatory pathway [42]. Interestingly, blockade of B7/CD28 costimulation abrogated the synergy between LPS and PD-L1 blockade in terms of CD8 T cell rescue (Fig 6B) and viral control (Fig 6C), demonstrating a B7/CD28 costimulation-dependent mechanism. It is important to clarify that B7 blockade did not affect APC numbers or subset distribution in spleen, but PD-1 therapy significantly reduced the frequencies of myeloid DCs (Fig 6D–6G).

### DC-intrinsic TLR4 triggering is necessary for improving PD-1 therapy after LPS stimulation

**Fig 7:**
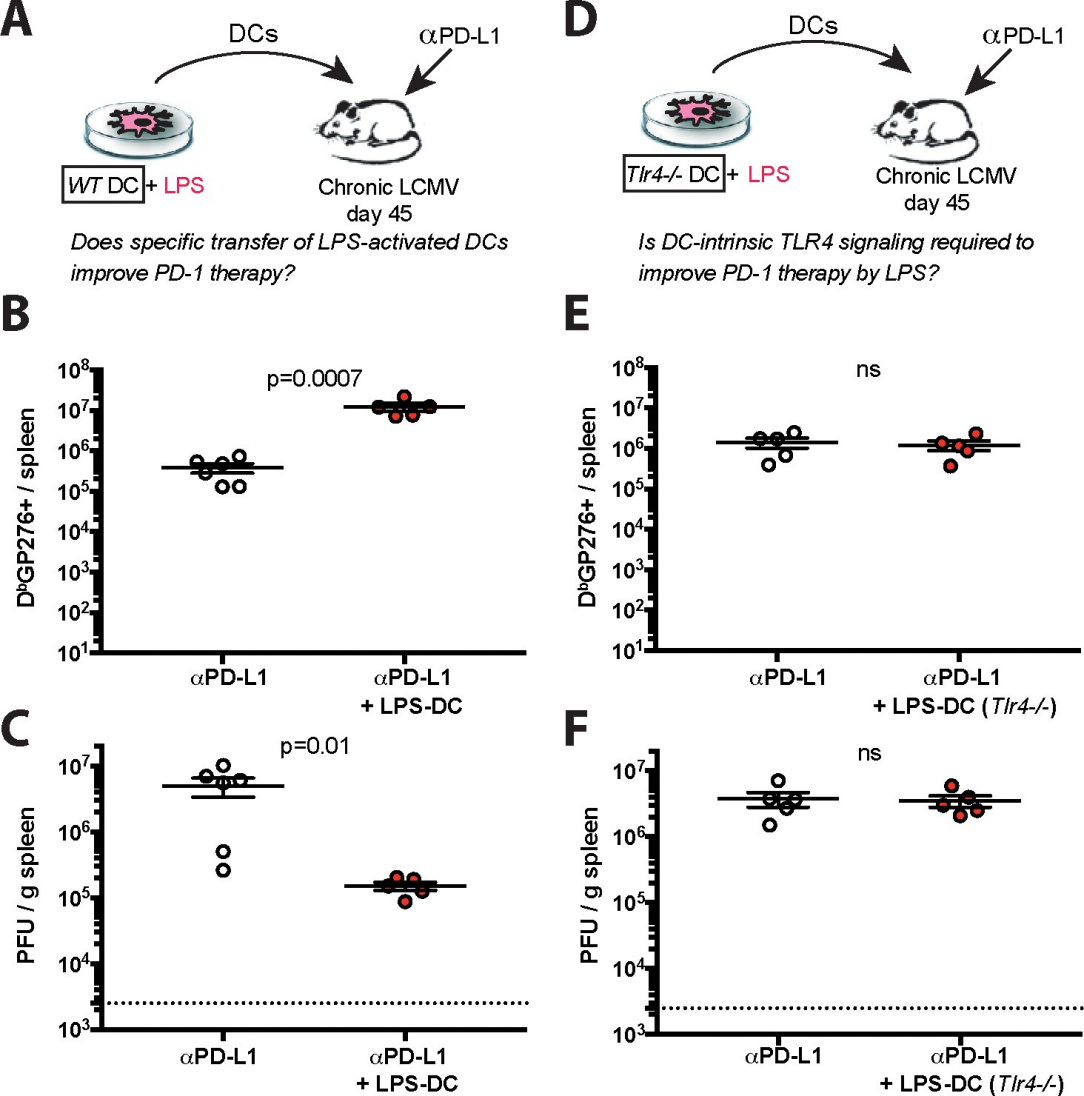
LPS-activated DCs also improve PD-1 therapy. (**A**) Experimental outline for evaluating whether LPS-activated DCs can improve the efficacy of PD-L1 blockade therapy. Mice chronically infected with LCMV Cl-13 received a PD-L1 blockade regimen combined with specific transfer of LPS-activated DCs. (**B**) Summary of virus-specific CD8 T cells in spleen. (**C**) Summary of viral control in spleen. (**D**) Experimental outline for evaluating the role of TLR4 signaling in potentiating the efficacy of PD-L1 blockade therapy after LPS treatment. Mice chronically infected with LCMV Cl-13 received PD-L1 blockade with LPS-activated DCs that lacked TLR4 (*Tlr4-/-*). (**E**) Summary of virus-specific CD8 T cells in spleen. (**F**) Summary of viral control in spleen. For spleen plaque assays, the limit of detection is indicated by a dashed line. Data are from one representative experiment. Experiments were performed 2 times with similar results, n = 4–6 mice per experiment; data from one experiment are shown. ns, not significant. Indicated p-values were calculated using Mann-Whitney tests. Error bars represent SEM.

To evaluate whether DCs mediated the synergistic effects of LPS on PD-1 therapy, we generated bone marrow derived DCs from infected mice, and stimulated these cells *ex vivo* with LPS for 24 hours, followed by extensive washing to remove LPS. We adoptively transferred 10^7^ DCs into chronically infected mice, followed by PD-L1 blockade (Fig 7A). Injection of LPS-activated DCs resulted in greater improvement of CD8 T cells, relative to PD-L1 blockade alone (Fig 7B). Accordingly, antiviral control was also improved by this combined therapy (Fig 7C). No effect was observed by transferring LPS-activated DCs alone (S8 Fig). Altogether, these data demonstrate a DC-intrinsic mechanism for the synergy.

TLR4 is a receptor for LPS, but various intracellular molecules can also sense LPS and mediate potent inflammatory responses [43–45]. To ascertain if TLR4 was critical for the potentiation of PD-1 therapy by LPS, we repeated the above experiment, but using *Tlr4*-/- DCs (Fig 7D). However, *Tlr4-/-* DCs did not improve PD-1 therapy (Fig 7E and 7F). We compared the phenotype of wild type and *Tlr4-/-* DC responses following LPS stimulation. Wild type DCs exhibited profound phenotypic and morphological changes upon LPS stimulation, but this was not observed in *Tlr4-/-* DCs (S9A and S9B Fig). Taken together, these data indicate that the synergistic effect of LPS is mediated strictly by a TLR4-dependent mechanism and not by alternative LPS sensors.

### Transfer of LPS-activated DCs to improve PD-1 therapy is well tolerated

We showed that systemic LPS administration improves PD-1 therapy, but systemic LPS administration resulted in wasting disease. Even at our low sublethal dose (25 μg), mice exhibited hunched posture and lethargy within 24 hours of systemic LPS treatment. In addition, systemic LPS administration resulted in significant weight loss (S10A Fig). On the other hand, the specific transfer of DCs activated *ex vivo* with LPS was safer and did not induce overt wasting (S10B Fig). These data demonstrate that adoptive transfer of LPS-activated DCs was substantially safer compared to systemic LPS administration.

### PD-1 therapy is not dependent on TLR4 signaling

The prior experiments demonstrate that TLR4/LPS signaling improves the efficacy of PD-1 therapy, but an unanswered question is if TLR4 is mechanistically required for PD-1 therapy. To answer this question, we infected wild type or *Tlr4-/-* mice with chronic LCMV, and at day 45 post-infection, mice received PD-L1 blocking antibodies (S11A Fig). Both *Tlr4-/-* and wild type mice exhibited improvement in CD8 T cells (S11B Fig) and enhanced viral control (S11C Fig) following PD-L1 blockade, demonstrating that TLR4 is dispensable during PD-L1 blockade therapy.

We then interrogated whether LPS could improve PD-1 therapy in *Tlr4*-/- mice. Importantly, we demonstrate that LPS treatment in *Tlr4-/-* mice does not improve PD-1 therapy (S12 Fig). Collectively, these experiments highlight an important distinction: The TLR4 pathway can improve PD-1 therapy, but the TLR4 pathway is not mechanistically required for PD-1 therapy to rescue CD8 T cells.

## Discussion

CD8 T cell exhaustion is a hallmark of chronic viral infections, such as HIV and HCV, which kill millions of people every year. CD8 T cells are critical for controlling chronic viral infections, but they become exhausted and unable to clear the persistent antigen due to upregulation of inhibitory molecules, including PD-1. Blockade of either the receptor (PD-1) or the ligand (PD-L1) can rescue exhausted CD8 T cells during chronic infection, but these therapies have clinical limitations that are not well-understood. Recent reports using cancer models have shown intriguing associations between gut microbiota and the host response to PD-1 therapy, but the specific microbial products that improve responses remain unknown [13, 14]. Our data using the chronic LCMV model demonstrate a potent synergism between bacterial LPS (a ubiquitous component of the microbiome) and PD-1 therapy. Treatment of mice with sublethal doses of LPS followed by PD-L1 blockade resulted in a more striking CD8 T cell rescue relative to PD-L1 blockade alone, identifying for the first time a specific bacterial product that affects PD-1 therapy. It is important to highlight that the effects of combined LPS and PD-1 therapy are not additive, but synergistic, as LPS alone did not induce rescue of exhausted CD8 T cell responses. Such synergy was surprising and difficult to theoretically predict based on prior studies with LPS treatment alone.

Systemic LPS administration potentiated PD-1 therapy, but this adjuvant also caused excessive weight loss and inflammation. In light of this, we aimed to develop a safer approach by stimulating DCs *ex vivo* with LPS, followed by extensive washing to remove LPS prior to adoptive transfer. LPS-activated DCs also synergized with PD-1 therapy and were substantially safer. DC immunotherapy has been used previously in clinical trials to revert CD8 T cell exhaustion, but it has not shown significant clinical benefits [46–49]. Our studies are novel, because we show for the first time that DC immunotherapy using LPS-activated DCs improves the efficacy of PD-1 therapy. LPS is one of the most immunogenic adjuvants ever discovered, and LPS treatment induces potent activation of naïve and memory CD8 T cells [18, 50, 51], but the effect of LPS on exhausted CD8 T cells has remained understudied. Our initial prediction, based on those prior reports, was that LPS alone would rescue exhausted CD8 T cells, but this was not the case. Such result could be explained by the PD-1/PD-L1 pathway. LPS not only upregulates costimulatory B7, but it also upregulates inhibitory PD-L1, and since exhausted CD8 T cells overexpress PD-1, they are more susceptible to PD-1/PD-L1 mediated inhibition relative to naïve or memory CD8 T cells. Taken together, both costimulatory and inhibitory ligands are co-regulated by LPS/TLR4 signaling, and thus, LPS/TLR4 signaling can only improve exhausted CD8 T cells when inhibitory PD-L1 signals are concomitantly blocked. B7/CD28 costimulation is required for CD8 T cell rescue after PD-1 blockade [42, 52], but during chronic viral infection, B7 expression by DCs is limited, resulting in decreased costimulation of exhausted CD8 T cells [27]. Therefore, B7/CD28 costimulation is a critical rate-limiting step that determines the success of PD-1 therapies, and we demonstrate that it is plausible to reinforce B7/CD28 costimulation during chronic viral infection by triggering TLR4 with LPS, rendering exhausted CD8 T cells more responsive to PD-1 therapy. Altogether, we demonstrate that the synergy between microbial LPS and PD-1 therapy is dependent on TLR4 signaling and B7/CD28 costimulation. We also demonstrate that this synergy is at least partially due to DC-intrinsic effects, but we cannot rule out the contribution of other APC subsets. Moreover, we show that the synergy is not dependent on IFN-I responses and does not seem to be caused by attenuated Treg responses.

A critical mechanistic insight from our studies is that the TLR4 pathway improves PD-1 therapy, but it is not itself required for PD-1 therapy. This is an important distinction, since a fraction of humans has genetic deficiencies in the TLR4 pathway, which results in a plethora of opportunistic infections [53, 54]. Our studies suggest that PD-1 therapy could still be effective in these patients. TLR4 triggering with LPS also upregulated MHC on DCs, but dissecting the role of MHC is technically difficult, since exhausted CD8 T cells require continuous TCR recognition to persist *in vivo*, a process colloquially referred to as “antigen-addiction” [55]. We also reason that during chronic viral infection, CD8 T cells are not critically regulated by limited MHC/TCR interactions, since the persistent antigen is expressed and presented ubiquitously.

The discovery of PD-1 therapies to rescue exhausted virus-specific CD8 T cells was initially made using the chronic LCMV model [7]. PD-1 therapies were later demonstrated to also improve exhausted CD8 T cells in other models of chronic viral infection [1–6]. However, PD-1 therapies have not been yet licensed to treat chronic viral infection, because of their limited capacity to induce clinically relevant antiviral control. In light of this, our data make a compelling case for evaluating whether TLR4 signaling can improve PD-1 therapy in other models of chronic viral infection.

Interestingly, low LPS levels can be detected in plasma under normal conditions, and the translocation of LPS from the intestinal microbiota to the circulation increases during chronic infection [56–58]. Moreover, injection of low LPS doses has been shown to be safe in humans, suggesting that there is a threshold of tolerance to this naturally occurring molecule, with the maximum tolerated dose in humans shown to be ~4 ng/kg [59, 60]. Future studies will identify whether this safe dose of LPS is still sufficient to improve PD-1 therapy in humans, and whether plasma or intestinal LPS levels could serve as biomarkers to predict the efficacy of PD-1 therapy. Regimens that reinforce B7/CD28 costimulation on exhausted CD8 T cells using CD28 agonistic antibodies or recombinant B7 proteins may also be of interest to improve PD-1 therapies.

In summary, our study has various novel findings. We show that exhausted CD8 T cells cannot be re-activated by LPS alone, but LPS sensitizes exhausted CD8 T cells to respond more vigorously to PD-1 therapy. We also show for the first time a specific microbial product that augments the efficacy of PD-1 therapy. These findings could have important implications for the development of novel treatments for chronic viral infections, and for understanding how natural components of the microbiome affect responses to immune checkpoint therapies.

## Materials and methods

### Mice, infections and treatments

6-8-week old female and male C57BL/6 wild type mice were used in all experiments. We also used *Tlr4-/-* mice in the C57BL/6 background. All mice were from Jackson laboratories. All infections were intravenous (i.v.) via the lateral tail vein and using a mouse restrainer. Mice were infected with 2x10^6^ PFU of LCMV Cl-13. Before starting treatments, we randomized mice in terms of viremia and number of virus-specific (D^b^GP276+) cells in PBMCs. All Antibodies for *in vivo* treatments were purchased from BioXCell, and were diluted in sterile PBS. To induce lifelong uncontrolled multiorgan infection, CD4 T cells were depleted at the time of infection with 500 μg of a CD4 depleting antibody (GK1.5) administered intraperitoneally (i.p.), as described previously [61]. PD-L1 blocking antibodies (10F.9G2) were administered i.p. at 200 μg, every three days, five times, as previously shown [7]. B7.1 and B7.2 blocking antibodies (16-10A1 and GL-1, respectively) were administered at 500 μg (250 μg each), every three days, five times. IFNAR1 blocking antibodies (MAR1-5A3) were administered at 500 μg, every three days, five times. This MAR1-5A3 antibody binds to interferon α/β receptor subunit 1 (IFNAR1) and blocks binding to interferons α/β, abrogating the induction of ISGs *in vivo* [22–26]. IgG isotype controls were used in all experiments. The first dose of B7 and IFNAR1 blocking antibodies were administered at least 6 hours before the first injection of LPS and PD-L1 blocking antibodies. Sublethal LPS doses were administered i.p. at 25 μg per mouse, on days 0 and 7 of the PD-L1 blockade regimen. Other TLR4 agonists were also administrated at 25 μg per mouse, DCs were harvested and activated *in vitro* as shown before [62, 63]. In brief, bone marrow cells from infection-matched mice were cultured for 5 days in GM-CSF (Sigma) at 20 ng/mL, and stimulated for 1 day with 100 ng/mL of *E*. *coli* derived LPS (Sigma). DCs were washed 5 times prior to injection to remove traces of LPS, and 10^7^ DCs were injected i.v. at day 0 and 7 of the PD-L1 blockade regimen.

### Quantification of viral titers

Quantification of LCMV titers was performed on Vero E6 cell monolayers as previously described [64]. In brief, Vero E6 cells (ATCC) were seeded onto 6-well plates, and once they reached ~95% confluency, the media was removed and 200 μL of serial viral dilutions were slowly pipetted on top of the monolayers. Plates were rocked every 10 min in a 37°C, 5% CO_2_ incubator. After 1 hr, 200 μL of media was aspirated out of each well, and the monolayers were overlaid with a 1:1 mixture of 2x199 media and 1% agarose. After 4 days of culture in a 37°C, 5% CO_2_ incubator, a second overlay was added, consisting of a 1:1 solution of 2x199 media and 1% agarose and 1:50 of neutral red dye. Overlay was removed with forceps on day 5 and plaques were counted using a transluminator. All mouse experiments were performed with approval of the NU Institutional Animal Care and Use Committee (IACUC).

### ELISA

ELISA to measure LCMV‐specific IgG responses in sera was performed using lysates of BHK‐21 cells infected with LCMV Cl-13. ELISA plates (Nunc MaxiSorp, 439454) were coated with infected BHK-21 lysates for 48 hr at room temperature. Twelve serial 30-fold dilutions of sera were added onto each well, followed by incubation with goat anti-mouse IgG HRP (SouthernBiotech, 1030–05). Sure Blue TMB peroxidase (KLP, 52-00-03) was added onto each well, and after 8 minutes, TMB Stop solution (KLP, 50-85-06) was added. ELISA plates were immediately read at 490 nm in a Spectramax 384 plus (Molecular Devices).

### Reagents, flow cytometry and antibodies for *in vitro* experiments

Single cell suspensions were obtained from PBMCs and various tissues as previously described [65]. Live cells were gated using Live/Dead fixable dead cell stain (Invitrogen). LCMV MHC class I tetramers were obtained from the NIH tetramer facility (Emory University). Cells were stained with anti- CD8α (53–6.7), -CD44 (IM7), -Granzyme B (MHGB04), -Ki67 (B56), -PD-L1 (MIH5), -B7.1 (16-10A1), -B7.2 (GL-1). Anti-mouse flow cytometry antibodies were purchased from BD Pharmingen, except for CD44 (Biolegend) and Granzyme B (Invitrogen). Flow cytometry samples were acquired with a Becton Dickinson LSRII and analyzed using FlowJo (Treestar). For confocal microscopy, spleen OCT sections were stained with rat anti-mouse PD-L1 (10F.9G2) at 1:200 dilution from a 2 mg/mL stock. Slides were then stained with a secondary anti-rat IgG antibody conjugated to Cy3. Microscopy slides were acquired using an Evos digital inverted microscope (Advanced Microscopy Group).

### RNA-seq data acquisition and analysis

Gene expression profiling was performed as described previously [66, 67]. In brief, splenic CD8 T cells were MACS-sorted at day 15 after treatment, using a MACS negative selection kit (STEMCELL). Purified CD8 T cells were stained with D^b^GP276 tetramer, live dead stain, and flow cytometry antibodies for CD8 and the CD44 activation marker. ~20,000 live, CD8+, CD44+, D^b^GP276+ cells were FACS-sorted to approximately 97% purity using a FACS Aria (BD Biosciences). FACS-sorted cells were collected in 10% FBS RPMI, and were then spun at 2000 rpm for 10 minutes at 4°C. The supernatant was aspirated, and cell pellets were resuspended in 1 mL of TRIzol (Life Sciences) inside a fume hood. All samples were stored at -80°C. The next day, RNA extraction was performed using the RNAdvance Tissue Isolation kit (Agencourt) on a plate magnet, following the manufacturer’s instructions. RNA quality assessment and HiSeq sequencing (Illumina) were performed at the NUSeq core at Northwestern University (Chicago, IL). Revigo pathway analyses were generated by pasting ranked genes in the **G**ene **O**ntology en**RI**chment ana**L**ysis and visua**L**iz**A**tion (Gorilla) tool [68, 69], and the output was plotted as GO terms to generate ranked cellular pathways [70]. Ingenuity Pathway Analyses (IPA) were performed using the Molecular Activity Predictor software (Qiagen). Gene Set Enrichment Analysis (GSEA) were performed with GSEA (Broad Institute) using C7 databases. Data included 3 control mice, 3 mice treated with PD-L1 blockade, and 4 mice treated with LPS and PD-L1 blockade. RNA-Seq data were deposited in the GEO database (GSE123153), titled “Gene expression comparison of exhausted CD8 T cells after PD-L1 blockade alone or PD-L1 blockade combined with LPS,” at https://www.ncbi.nlm.nih.gov/geo/query/acc.cgi?acc=GSE123153.

### Statistical analysis

Statistical analyses were performed using the test indicated in each figure legend. Data were analyzed using Prism software (Graphpad). Statistical significance was established at p≤0.05.

### Ethics statement

Mouse studies were reviewed and approved by the Institutional Animal Care and Use Committee (IACUC) at Northwestern University (protocol number IS00003258, IS00008785, IS00003324). All mouse experiments were performed minimizing distress. Mouse experiments were conducted in accordance with recommendations listed in the Guide for the Care and Use of Laboratory Animals of the NIH.

## Supporting information

S1 Fig Revigo analyses of rescued CD8 T cells. Data were analyzed in Revigo software utilizing GO terms that exhibited enrichment in GOrilla. Most enriched pathways represented metabolism, mitosis, and immune related pathways, which were highest in virus-specific CD8 T cells following combined therapy. Data from one experiment are shown. RNA-Seq data are from PD-L1 therapy alone (n = 3), or combined LPS and PD-L1 therapy (n = 4) at day 15 post-treatment, as shown in Fig 4A.
(TIF)

S2 Fig Ingenuity Pathway Analysis (IPA) showing the top cellular pathways enriched in virus-specific CD8 T cells following combined therapy. Log (p-value) ranked pathways are shown. Blue shading represents a negative z-score, orange shading represents a positive z-score, white shading represents a z-score of 0, grey shading represents no activity pattern available. Data from one experiment are shown. RNA-Seq data are from PD-L1 therapy alone (n = 3), or combined LPS and PD-L1 therapy (n = 4) at day 15 post-treatment, as shown in Fig 4A.
(TIF)

S3 Fig Molecular Activity Predictor visualization showing enrichment in CD28 costimulation driven genes in virus-specific CD8 T cells following combined therapy. Overlay Molecule Activity Predictor (MAP) tool analyses of the CD28 costimulatory pathway. Data show canonical pathway for the genes in *CD28 Signaling in T Cells* dataset overlaid with hits from our RNA-Seq data. Significant gene pathway nodes are depicted by colored shading depending on their fold-change. White nodes indicate genes that were not detected, whereas grey indicates genes that were detected, but were not statistically significant. Colored double borders indicate that the molecule exhibits complexity. Refer to the legend panel on the right for additional information. Data from one experiment are shown. RNA-Seq data are from PD-L1 therapy alone (n = 3), or combined LPS and PD-L1 therapy (n = 4) at day 15 post-treatment, as shown in Fig 4A.
(TIF)

S4 Fig The IFNAR1 blocking antibody MAR1-5A3 abrogates the induction of IFN-I driven genes. (**A**) Representative FACS histograms showing the expression of PD-L1 and MHC-I following stimulation with IFNα. (**B**) Summary of PD-L1 expression after IFNα stimulation with or without IFNAR1 blocking antibody. (**C**) Summary of MHC-I expression after IFNα stimulation with or without IFNAR1 blocking antibody. 10^5^ CT26 cells were first incubated for 30 minutes with MAR1-5A3 or IgG (MOPC-21 isotype control) antibody. 500 IU of recombinant murine IFNα was added to the wells at 37°C for 24 hr. The following day, cells were washed with PBS, treated with accutase, and stained with antibodies against mouse PD-L1 and MHC-I. Data are pooled from different experiments. Experiments were performed twice, with 4–6 replicate wells per group. Indicated p-values used ANOVA for multiple comparisons with Holm-Sidak’s correction. Error bars represent SEM.
(TIF)

S5 Fig Phenotypic changes of splenic DCs following LPS treatment in chronically infected mice. (**A**) Summary of DC numbers. (**B**) Summary of MHC I expression. (**C**) Summary of MHC II expression. (**D**) Summary of B7.1 expression. (**E**) Summary of B7.2 expression. (**F**) Summary of B7.2 expression after treatment with various TLR agonists (MPLA, Monophosphoryl lipid A; LAM, Lipoarabinomannan). Only LPS can increase B7 expression on DCs of chronically infected mice. (**G**) Summary of PD-L1 expression. (**H**) PD-L1 expression by immunofluorescence of spleen. Spleen OCT sections were stained with an αPD-L1 antibody (10F.9G2), followed a secondary Cy3 labeled antibody. 40x magnification is shown. DCs were gated as live CD3- NK1.1- Ly6G- CD19- CD11c+. Chronically infected mice (day 45 post-infection) were injected with the indicated TLR agonist (25 μg) or a PBS control solution and sacrificed 24 hours after treatment to compare the phenotype of splenic DCs. Data are pooled from different experiments. Experiments were performed 3 times, n = 3–5 mice per experiment. Indicated p-values for all panels are calculated with Mann-Whitney tests, except for panel F, which used ANOVA for multiple comparisons with Holm-Sidak’s correction. Error bars represent SEM.
(TIF)

S6 Fig Phenotypic changes of other splenic APCs following LPS treatment in chronically infected mice. (**A**) Summary of MHC I expression on B cells. (**B**) Summary of B7.1 expression on B cells. (**C**) Summary of B7.2 expression on B cells. (**D**) Summary of PD-L1 expression on B cells. (**E**) Summary of MHC I expression on macrophages. (**F**) Summary of B7.1 expression on macrophages. (**G**) Summary of B7.2 expression on macrophages. (**H**) Summary of PD-L1 expression on macrophages. B cells were gated as live CD3- NK1.1- CD19+, and macrophages were gated as live CD3- NK1.1- CD19- F4/80+ CD11b+. Chronically infected mice (day 45 post-infection) were injected with LPS (25 μg) or a PBS control solution and sacrificed 24 hours after treatment to compare the phenotype of splenic B cells and macrophages. Data are pooled from different experiments. Experiments were performed 2 times, n = 3–5 mice per experiment. Indicated p-values for all panels are calculated with Mann-Whitney tests. Error bars represent SEM.
(TIF)

S7 Fig LPS induces high levels of costimulatory B7 and inhibitory PD-L1 molecules on DCs of naïve mice. (**A**) Summary of MHC I expression on DCs of naïve mice. (**B**) Summary of B7.1 expression on DCs of naïve mice. (**C**) Summary of B7.2 expression on DCs of naïve mice. (**D**) Summary of PD-L1 expression on DCs of naïve mice. DCs were gated as live CD3- NK1.1- Ly6G- CD19- CD11c+. Naïve mice were treated with the indicated TLR agonist (25 μg) or a PBS control solution and sacrificed 24 hours after treatment to compare the phenotype of splenic DCs. Data are pooled from different experiments. Experiments were performed 2 times, n = 3–5 mice per experiment. Indicated p-values for all panels are calculated with ANOVA for multiple comparisons with Holm-Sidak’s correction. Error bars represent SEM.
(TIF)

S8 Fig LPS-DCs alone do not significantly improve exhausted CD8 T cell responses. (**A**) Summary of virus-specific CD8 T cells in spleen. (**B**) Summary of viral control in spleen. Mice chronically infected with LCMV Cl-13 received LPS-activated DCs alone (without PD-L1 blocking antibodies). For spleen plaque assays, the limit of detection is indicated by a dashed line. Data are pooled from different experiments. Experiments were performed 2 times, n = 4 mice per experiment; ns, not significant. Indicated p-values were calculated using Mann-Whitney tests. Error bars represent SEM.
(TIF)

S9 Fig LPS-induced activation of DCs is TLR4-dependent. (**A**) Representative FACS histograms showing the expression of costimulatory B7 molecules following LPS stimulation in wild type or *Tlr4*-/- DCs. (**B**) LPS induces DC maturation in wild type, but not *Tlr4-/-* DCs. Maturation is evidenced by DC branching in wild type DCs stimulated with LPS. Red arrows indicate areas of dendrite branching 24 hours after LPS treatment; WT, wild type; 40X magnification is shown; scale bar represents 100 μm. DCs were harvested from chronically infected mice. Data are from one representative experiment. Experiments were performed 2 times with similar results, n = 4–5 mice per experiment.
(TIF)

S10 Fig Transfer of LPS-activated DCs does not induce wasting disease. (**A**) Summary of weight loss following systemic LPS administration. Experiment layout is identical to that of Fig 1A. (**B**) Summary of weight loss following specific transfer of LPS-activated DCs. Experiment layout is identical to that of Fig 7A. Data are pooled from different experiments. The experiments in panel A were performed 3 times, n = 3–5 mice per experiment; the experiments in panel B were performed 2 times, n = 4–5 mice per experiment. Indicated p-values compare pre- and post-treatment (day 14) weight for each group using Wilcoxon matched-pairs signed rank test; ns, not significant.
(TIF)

S11 Fig PD-1 therapy is not dependent on TLR4. (**A**) Experimental outline for evaluating whether PD-1 therapy is mechanistically dependent on TLR4 signaling. Wild type or *Tlr4-/-* mice that were chronically infected with LCMV Cl-13 (day 45 post-infection) received PD-L1 blockade therapy, and CD8 T cell responses and viral control were evaluated at day 15 post-treatment. (**B**) Absolute numbers of CD8 T cells in spleen producing IFN-γ after 5-hr stimulation with LCMV peptides (0.1 μg/mL) in the presence of brefeldin A and monensin at 37°C in 5% CO_2._ (**C**) Summary of viral control in spleen and lung. For plaque assays the limit of detection is indicated by a dashed line. Experiments were performed 2 times, n = 3–5 mice per experiment with similar results. Data from one representative experiment are shown. Indicated p-values compare control IgG versus αPD-L1, using Mann-Whitney tests. Error bars represent SEM.
(TIF)

S12 Fig LPS treatment in *Tlr4-/-* mice does not improve PD-1 therapy. (**A**) Experimental outline for evaluating whether TLR4 is necessary for LPS-induced improvement of PD-1 therapy. *Tlr4-/-* mice that were chronically infected with LCMV Cl-13 (day 45 post-infection) received PD-L1 blockade therapy, and CD8 T cell responses and viral control were evaluated at day 15 post-treatment. (**B**) Summary of D^b^GP276+ responses in spleen. (**C**) Summary of viral control in spleen. For plaque assays the limit of detection is indicated by a dashed line. Data are pooled from different experiments. Experiments were performed 2 times, n = 3–4 mice per experiment; ns, not significant. Statistical analyses were performed with ANOVA for multiple comparisons with Holm-Sidak’s correction. Error bars represent SEM.
(TIF)
