# Supplementary figures and images for: TLR4 signaling improves PD-1 blockade therapy during chronic viral infection
Source: PLoS Pathog. 2019 Feb 6;15(2):e1007583. doi: 10.1371/journal.ppat.1007583 (PMC6380600; doi:10.1371/journal.ppat.1007583)

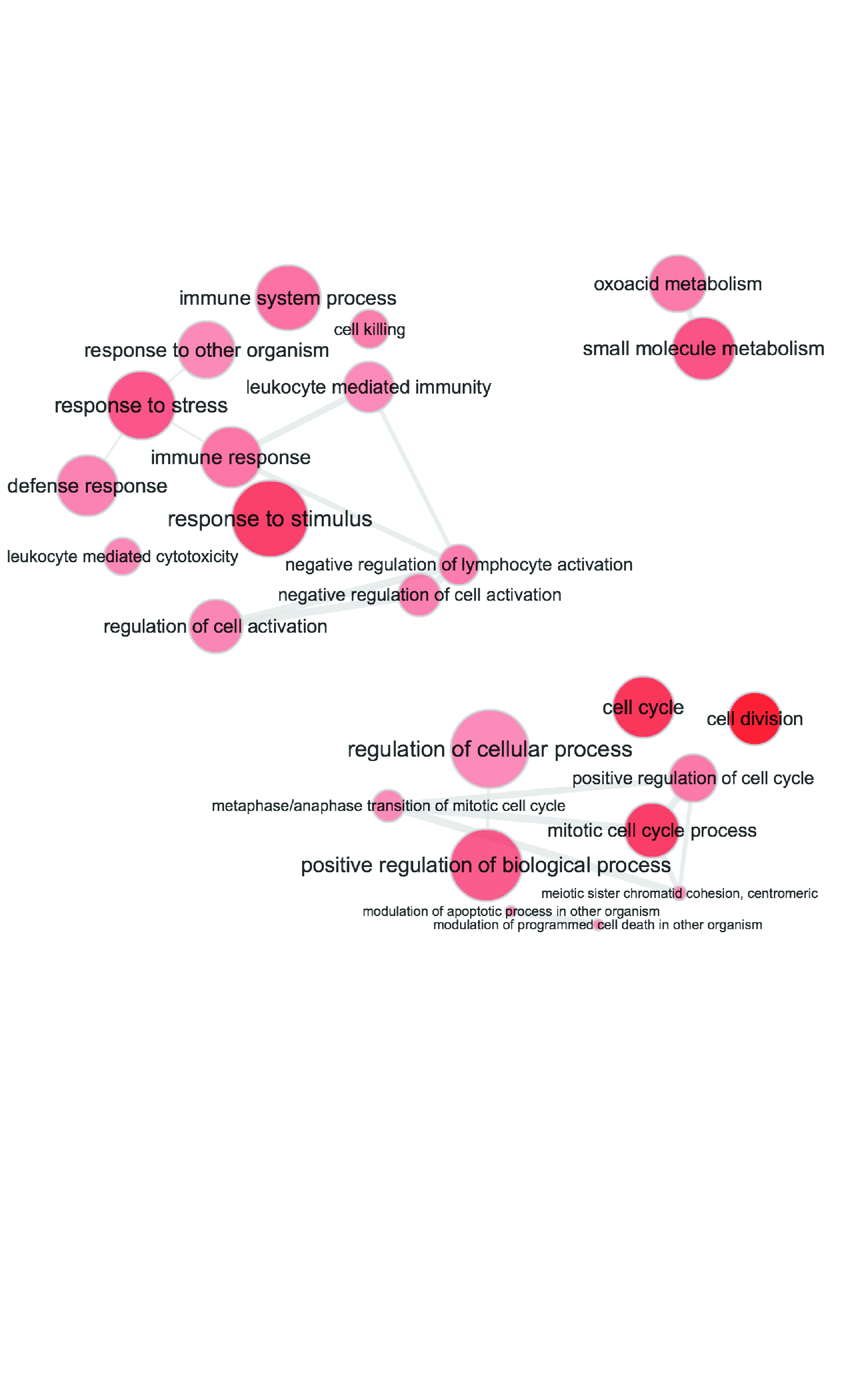

Supplement: S1 Fig — Data were analyzed in Revigo software utilizing GO terms that exhibited enrichment in GOrilla. Most enriched pathways represented metabolism, mitosis, and immune related pathways, which were highest in virus-specific CD8 T cells following combined therapy. Data from one experiment are shown. RNA-Seq data are from PD-L1 therapy alone (n = 3), or combined LPS and PD-L1 therapy (n = 4) at day 15 post-treatment, as shown in Fig 4A. (TIF) [file ppat.1007583.s001.tif]

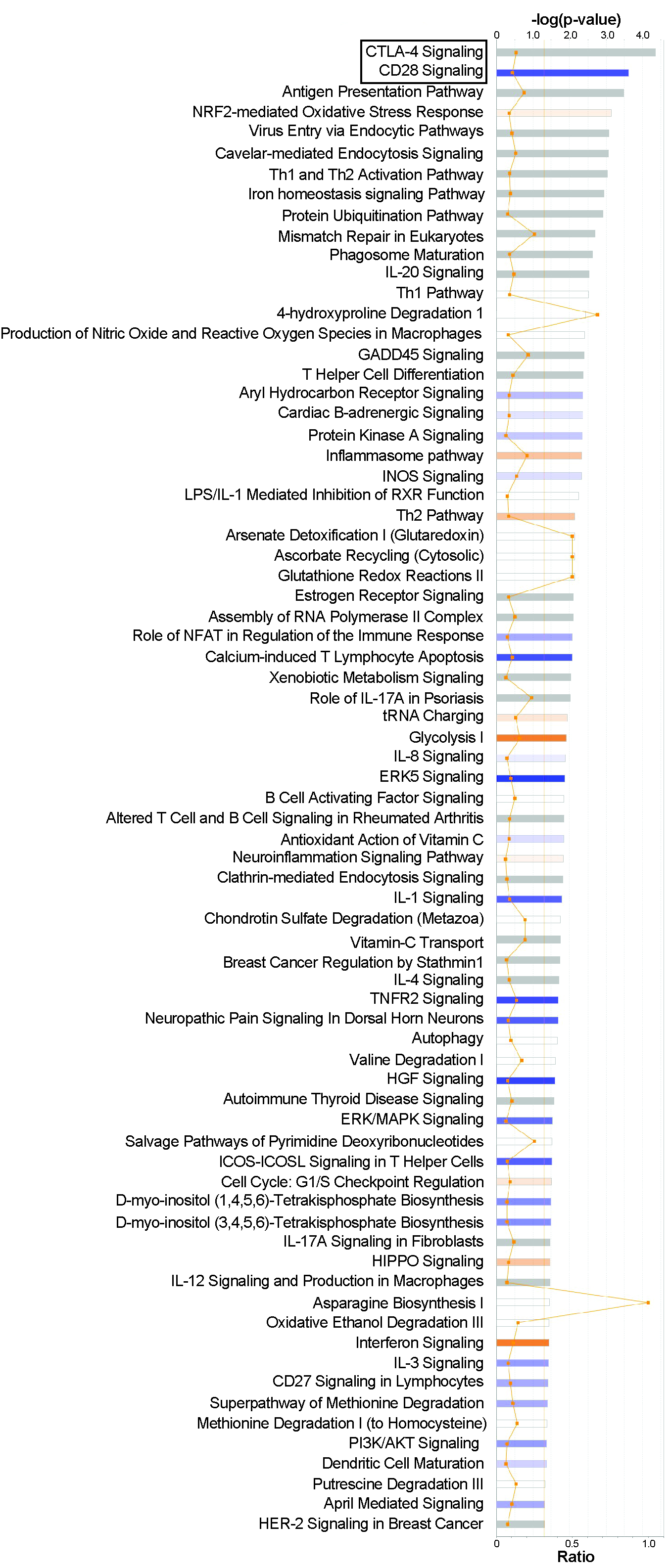

Supplement: S2 Fig — Log (p-value) ranked pathways are shown. Blue shading represents a negative z-score, orange shading represents a positive z-score, white shading represents a z-score of 0, grey shading represents no activity pattern available. Data from one experiment are shown. RNA-Seq data are from PD-L1 therapy alone (n = 3), or combined LPS and PD-L1 therapy (n = 4) at day 15 post-treatment, as shown in Fig 4A. (TIF) [file ppat.1007583.s002.tif]

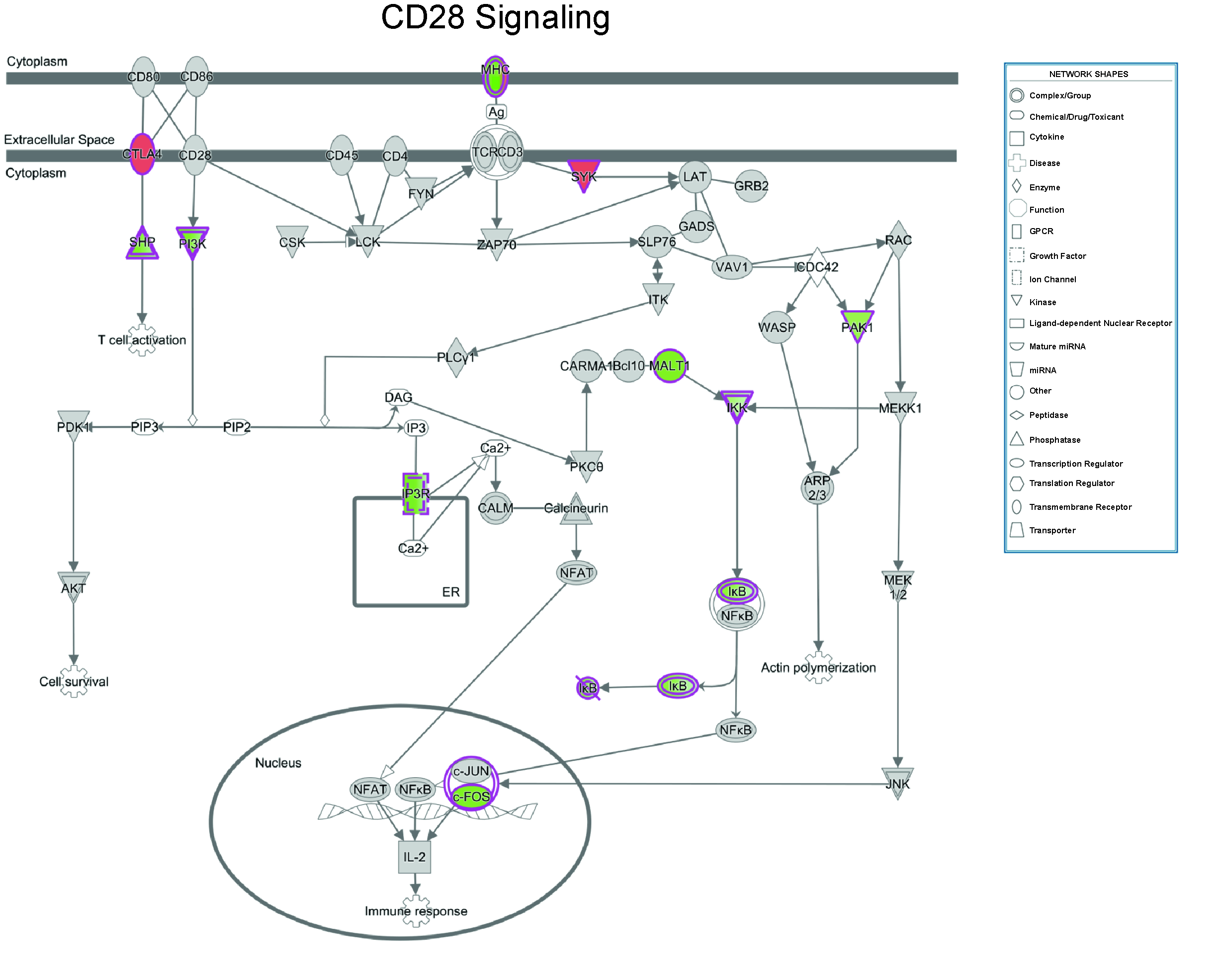

Supplement: S3 Fig — Overlay Molecule Activity Predictor (MAP) tool analyses of the CD28 costimulatory pathway. Data show canonical pathway for the genes in CD28 Signaling in T Cells dataset overlaid with hits from our RNA-Seq data. Significant gene pathway nodes are depicted by colored shading depending on their fold-change. White nodes indicate genes that were not detected, whereas grey indicates genes that were detected, but were not statistically significant. Colored double borders indicate that the molecule exhibits complexity. Refer to the legend panel on the right for additional information. Data from one experiment are shown. RNA-Seq data are from PD-L1 therapy alone (n = 3), or combined LPS and PD-L1 therapy (n = 4) at day 15 post-treatment, as shown in Fig 4A. (TIF) [file ppat.1007583.s003.tif]

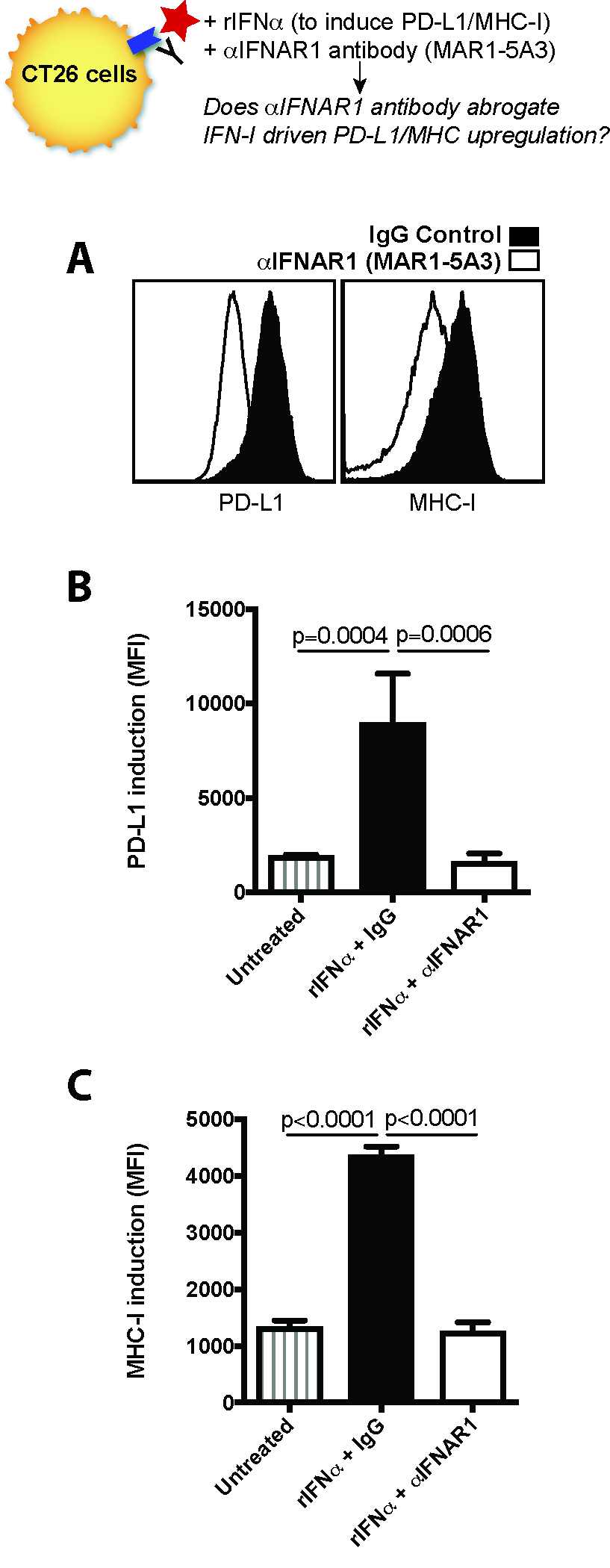

Supplement: S4 Fig — (A) Representative FACS histograms showing the expression of PD-L1 and MHC-I following stimulation with IFNα. (B) Summary of PD-L1 expression after IFNα stimulation with or without IFNAR1 blocking antibody. (C) Summary of MHC-I expression after IFNα stimulation with or without IFNAR1 blocking antibody. 105 CT26 cells were first incubated for 30 minutes with MAR1-5A3 or IgG (MOPC-21 isotype control) antibody. 500 IU of recombinant murine IFNα was added to the wells at 37°C for 24 hr. The following day, cells were washed with PBS, treated with accutase, and stained with antibodies against mouse PD-L1 and MHC-I. Data are pooled from different experiments. Experiments were performed twice, with 4–6 replicate wells per group. Indicated p-values used ANOVA for multiple comparisons with Holm-Sidak’s correction. Error bars represent SEM. (TIF) [file ppat.1007583.s004.tif]

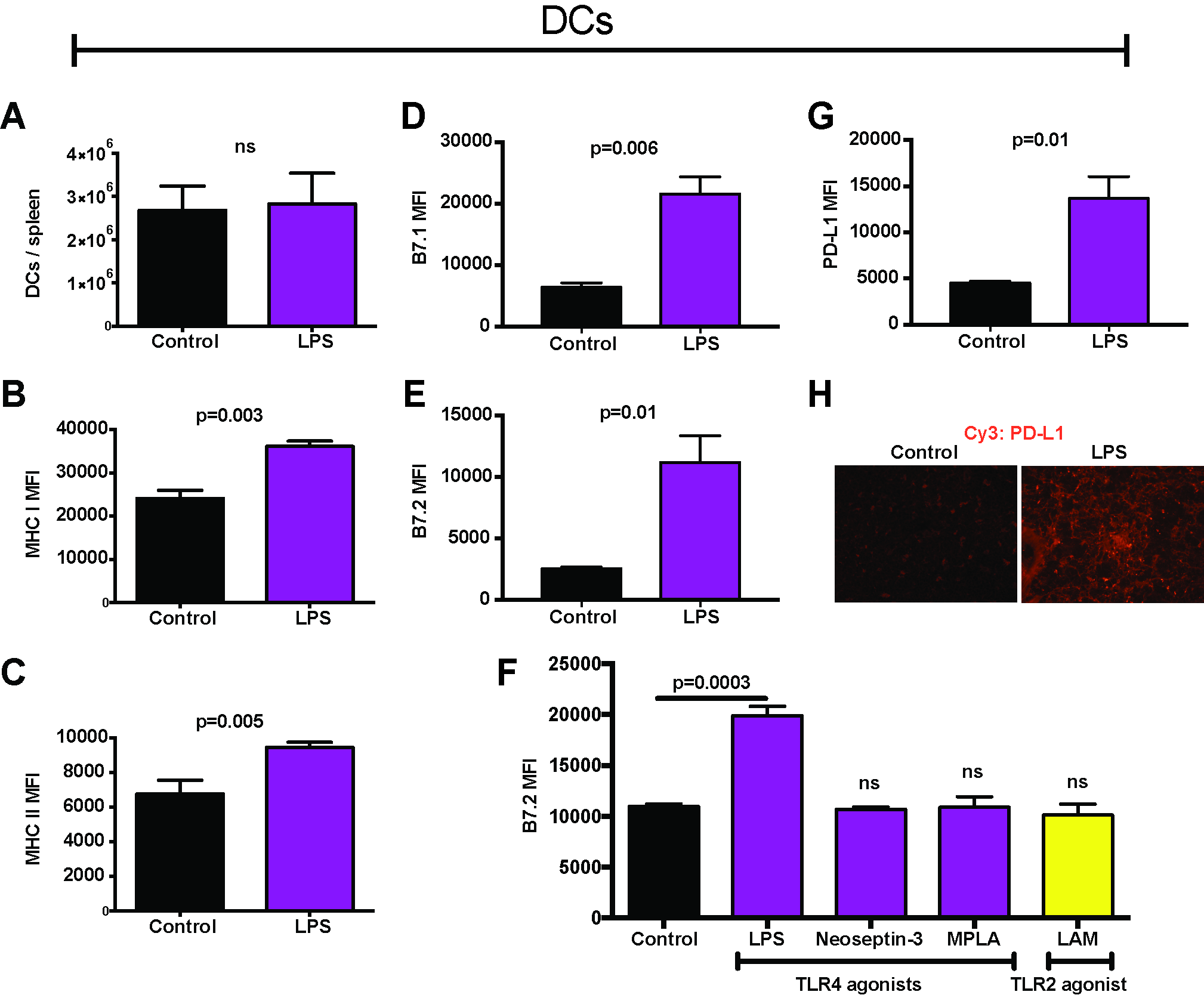

Supplement: S5 Fig — (A) Summary of DC numbers. (B) Summary of MHC I expression. (C) Summary of MHC II expression. (D) Summary of B7.1 expression. (E) Summary of B7.2 expression. (F) Summary of B7.2 expression after treatment with various TLR agonists (MPLA, Monophosphoryl lipid A; LAM, Lipoarabinomannan). Only LPS can increase B7 expression on DCs of chronically infected mice. (G) Summary of PD-L1 expression. (H) PD-L1 expression by immunofluorescence of spleen. Spleen OCT sections were stained with an αPD-L1 antibody (10F.9G2), followed a secondary Cy3 labeled antibody. 40x magnification is shown. DCs were gated as live CD3- NK1.1- Ly6G- CD19- CD11c+. Chronically infected mice (day 45 post-infection) were injected with the indicated TLR agonist (25 μg) or a PBS control solution and sacrificed 24 hours after treatment to compare the phenotype of splenic DCs. Data are pooled from different experiments. Experiments were performed 3 times, n = 3–5 mice per experiment. Indicated p-values for all panels are calculated with Mann-Whitney tests, except for panel F, which used ANOVA for multiple comparisons with Holm-Sidak’s correction. Error bars represent SEM. (TIF) [file ppat.1007583.s005.tif]

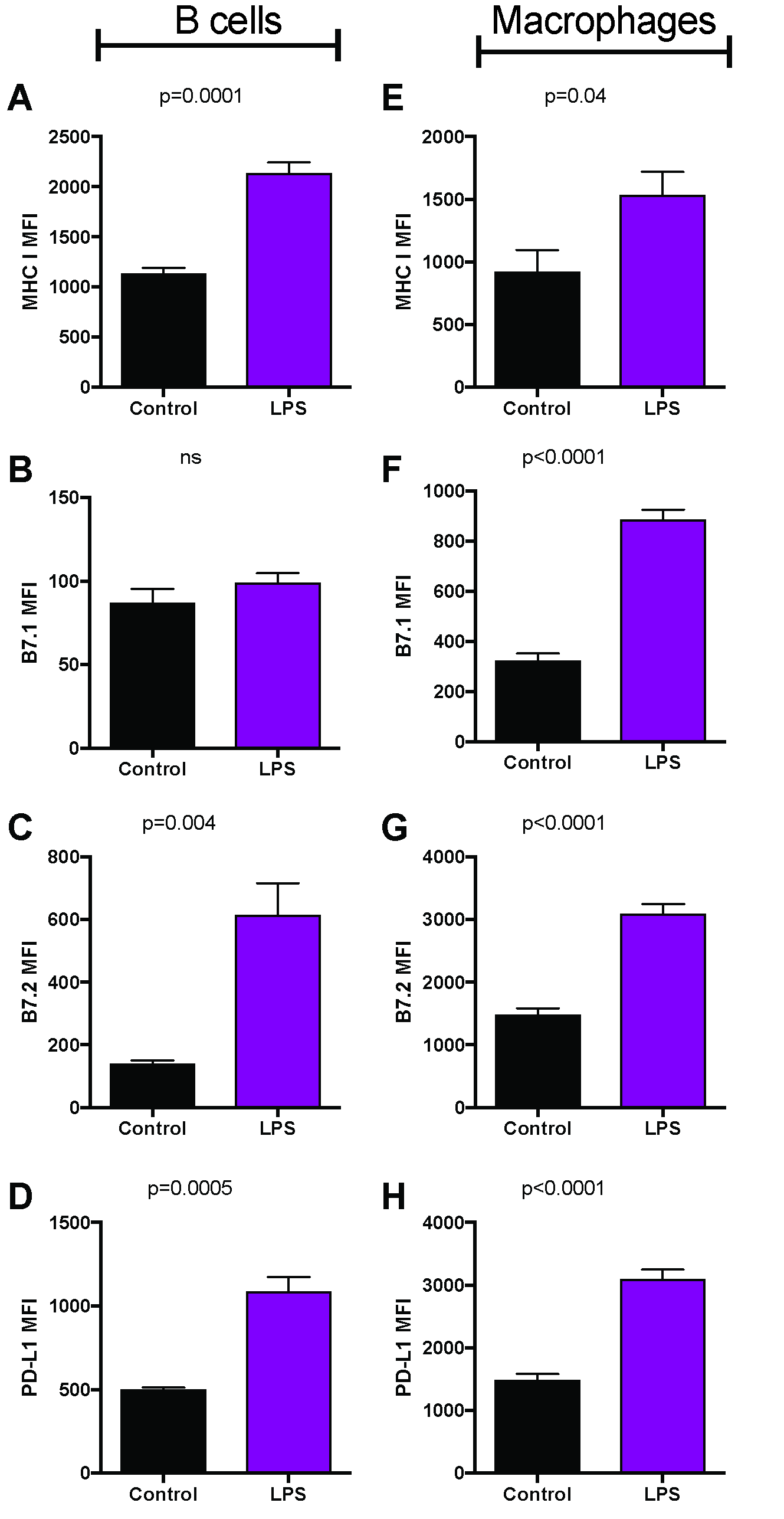

Supplement: S6 Fig — (A) Summary of MHC I expression on B cells. (B) Summary of B7.1 expression on B cells. (C) Summary of B7.2 expression on B cells. (D) Summary of PD-L1 expression on B cells. (E) Summary of MHC I expression on macrophages. (F) Summary of B7.1 expression on macrophages. (G) Summary of B7.2 expression on macrophages. (H) Summary of PD-L1 expression on macrophages. B cells were gated as live CD3- NK1.1- CD19+, and macrophages were gated as live CD3- NK1.1- CD19- F4/80+ CD11b+. Chronically infected mice (day 45 post-infection) were injected with LPS (25 μg) or a PBS control solution and sacrificed 24 hours after treatment to compare the phenotype of splenic B cells and macrophages. Data are pooled from different experiments. Experiments were performed 2 times, n = 3–5 mice per experiment. Indicated p-values for all panels are calculated with Mann-Whitney tests. Error bars represent SEM. (TIF) [file ppat.1007583.s006.tif]

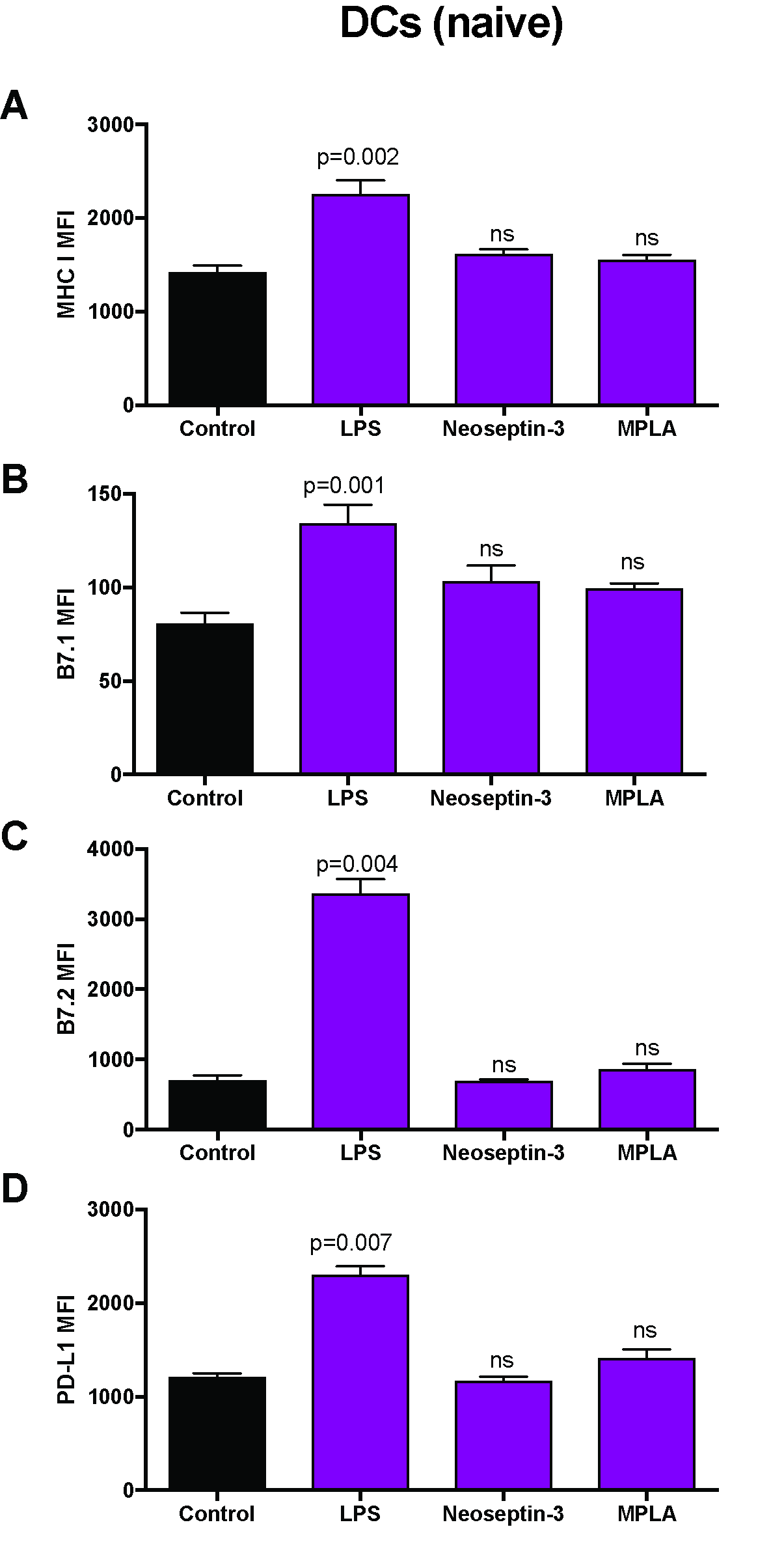

Supplement: S7 Fig — (A) Summary of MHC I expression on DCs of naïve mice. (B) Summary of B7.1 expression on DCs of naïve mice. (C) Summary of B7.2 expression on DCs of naïve mice. (D) Summary of PD-L1 expression on DCs of naïve mice. DCs were gated as live CD3- NK1.1- Ly6G- CD19- CD11c+. Naïve mice were treated with the indicated TLR agonist (25 μg) or a PBS control solution and sacrificed 24 hours after treatment to compare the phenotype of splenic DCs. Data are pooled from different experiments. Experiments were performed 2 times, n = 3–5 mice per experiment. Indicated p-values for all panels are calculated with ANOVA for multiple comparisons with Holm-Sidak’s correction. Error bars represent SEM. (TIF) [file ppat.1007583.s007.tif]

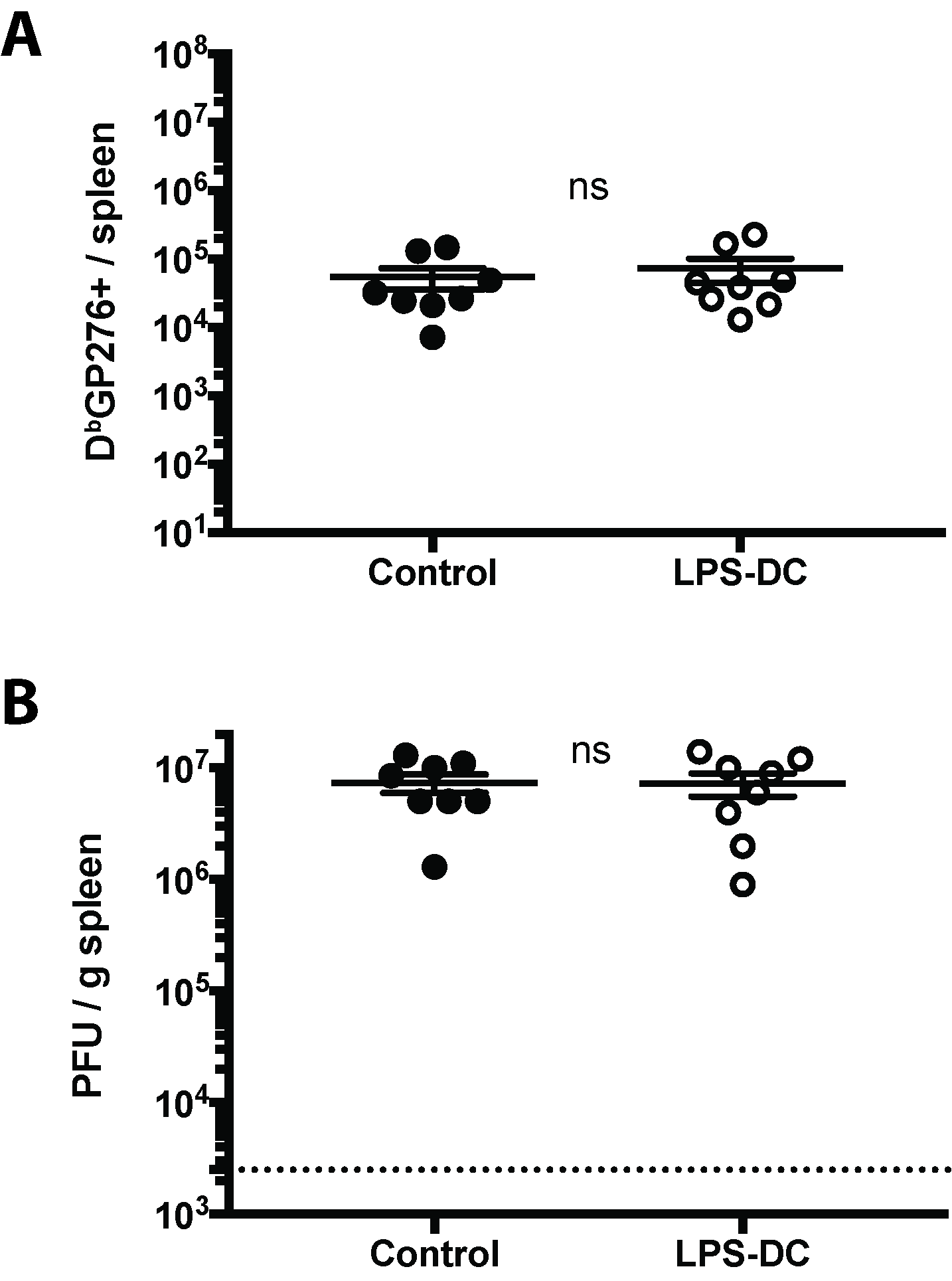

Supplement: S8 Fig — (A) Summary of virus-specific CD8 T cells in spleen. (B) Summary of viral control in spleen. Mice chronically infected with LCMV Cl-13 received LPS-activated DCs alone (without PD-L1 blocking antibodies). For spleen plaque assays, the limit of detection is indicated by a dashed line. Data are pooled from different experiments. Experiments were performed 2 times, n = 4 mice per experiment; ns, not significant. Indicated p-values were calculated using Mann-Whitney tests. Error bars represent SEM. (TIF) [file ppat.1007583.s008.tif]

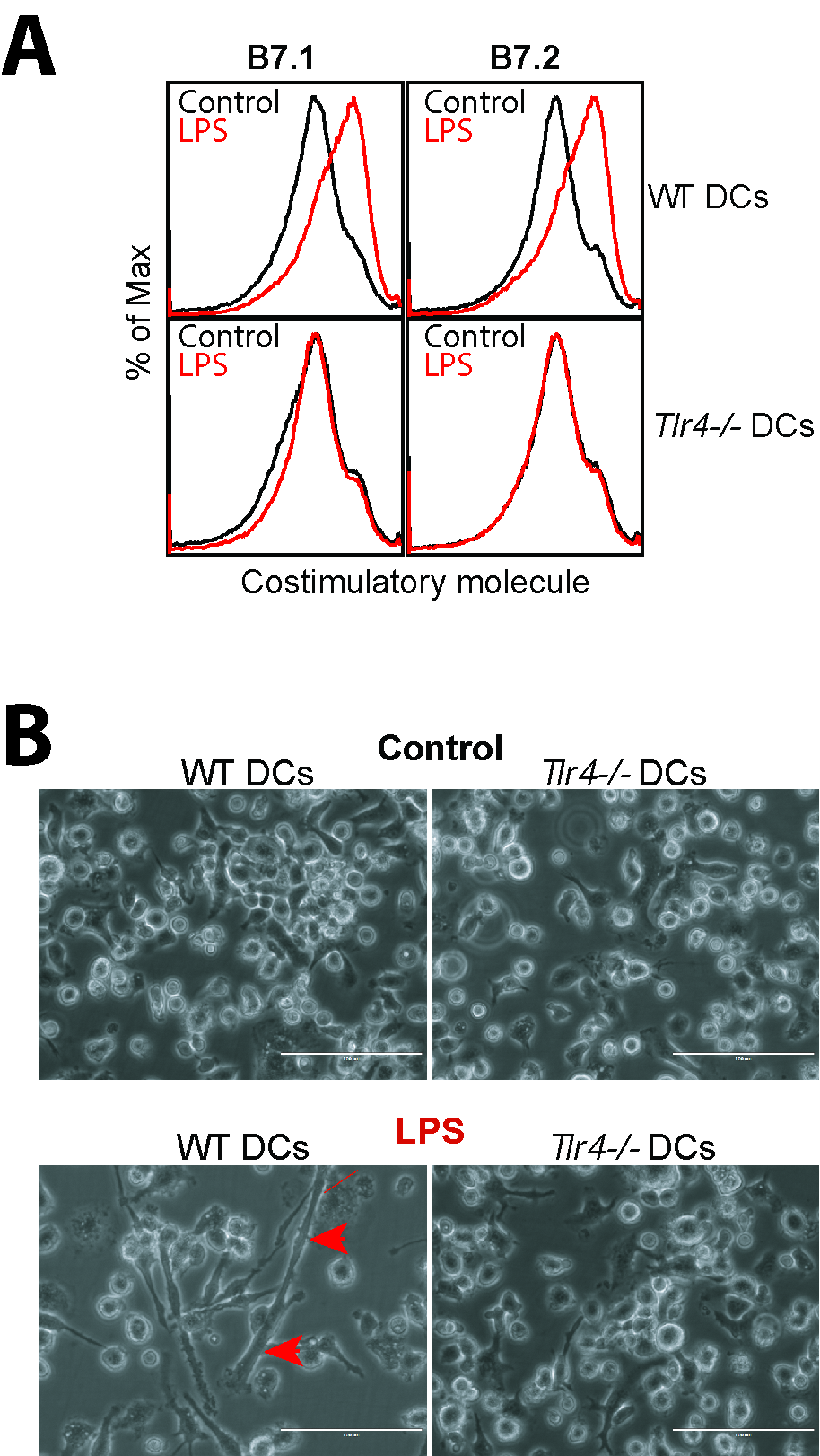

Supplement: S9 Fig — (A) Representative FACS histograms showing the expression of costimulatory B7 molecules following LPS stimulation in wild type or Tlr4-/- DCs. (B) LPS induces DC maturation in wild type, but not Tlr4-/- DCs. Maturation is evidenced by DC branching in wild type DCs stimulated with LPS. Red arrows indicate areas of dendrite branching 24 hours after LPS treatment; WT, wild type; 40X magnification is shown; scale bar represents 100 μm. DCs were harvested from chronically infected mice. Data are from one representative experiment. Experiments were performed 2 times with similar results, n = 4–5 mice per experiment. (TIF) [file ppat.1007583.s009.tif]

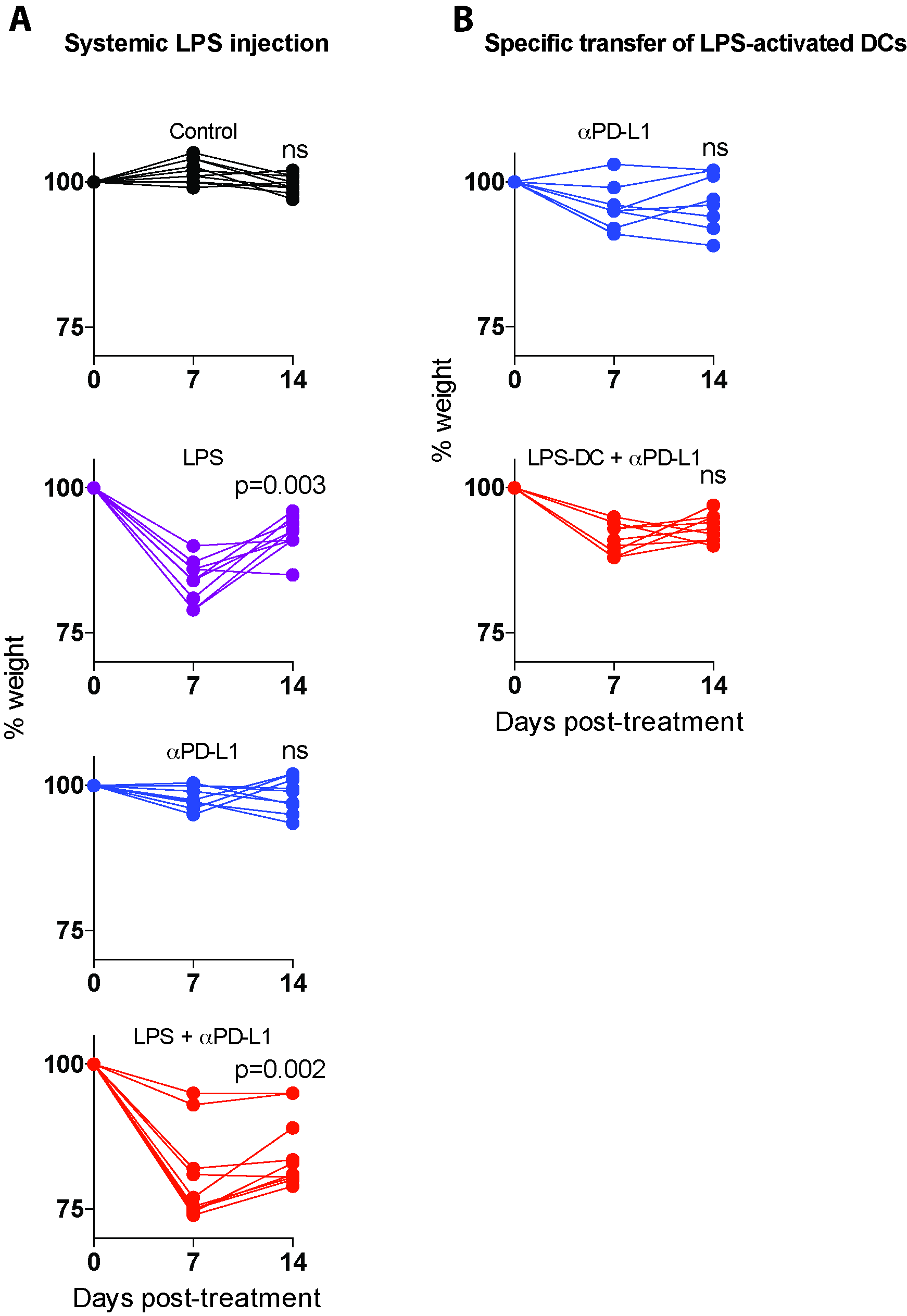

Supplement: S10 Fig — (A) Summary of weight loss following systemic LPS administration. Experiment layout is identical to that of Fig 1A. (B) Summary of weight loss following specific transfer of LPS-activated DCs. Experiment layout is identical to that of Fig 7A. Data are pooled from different experiments. The experiments in panel A were performed 3 times, n = 3–5 mice per experiment; the experiments in panel B were performed 2 times, n = 4–5 mice per experiment. Indicated p-values compare pre- and post-treatment (day 14) weight for each group using Wilcoxon matched-pairs signed rank test; ns, not significant. (TIF) [file ppat.1007583.s010.tif]

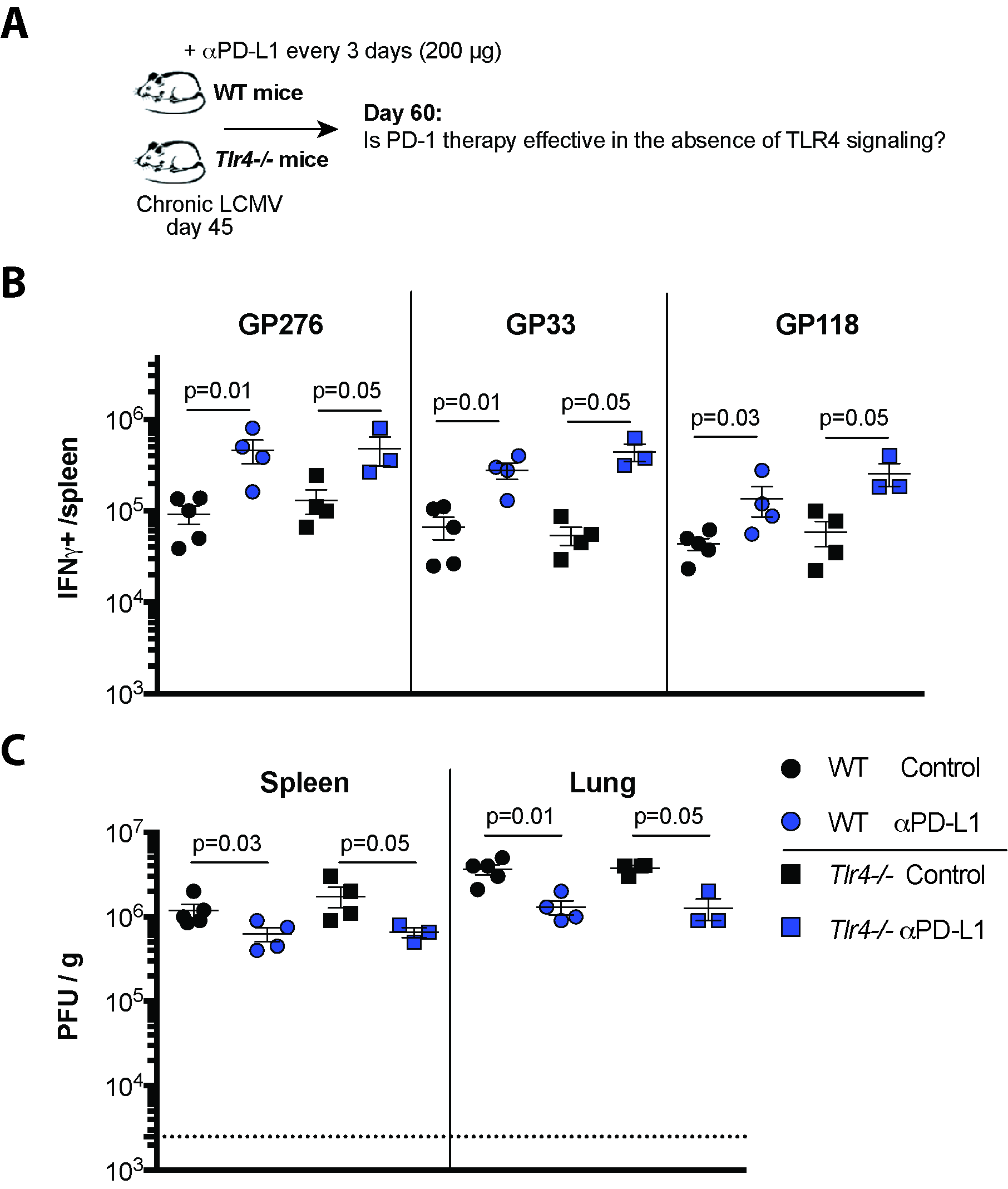

Supplement: S11 Fig — (A) Experimental outline for evaluating whether PD-1 therapy is mechanistically dependent on TLR4 signaling. Wild type or Tlr4-/- mice that were chronically infected with LCMV Cl-13 (day 45 post-infection) received PD-L1 blockade therapy, and CD8 T cell responses and viral control were evaluated at day 15 post-treatment. (B) Absolute numbers of CD8 T cells in spleen producing IFN-γ after 5-hr stimulation with LCMV peptides (0.1 μg/mL) in the presence of brefeldin A and monensin at 37°C in 5% CO2. (C) Summary of viral control in spleen and lung. For plaque assays the limit of detection is indicated by a dashed line. Experiments were performed 2 times, n = 3–5 mice per experiment with similar results. Data from one representative experiment are shown. Indicated p-values compare control IgG versus αPD-L1, using Mann-Whitney tests. Error bars represent SEM. (TIF) [file ppat.1007583.s011.tif]

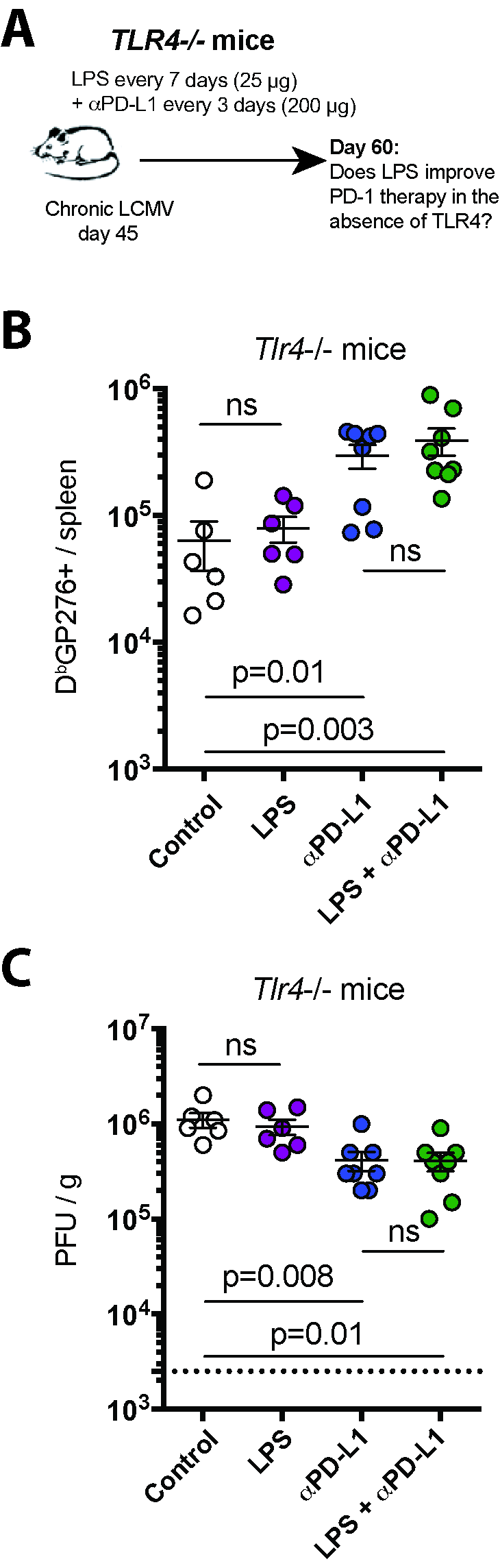

Supplement: S12 Fig — (A) Experimental outline for evaluating whether TLR4 is necessary for LPS-induced improvement of PD-1 therapy. Tlr4-/- mice that were chronically infected with LCMV Cl-13 (day 45 post-infection) received PD-L1 blockade therapy, and CD8 T cell responses and viral control were evaluated at day 15 post-treatment. (B) Summary of DbGP276+ responses in spleen. (C) Summary of viral control in spleen. For plaque assays the limit of detection is indicated by a dashed line. Data are pooled from different experiments. Experiments were performed 2 times, n = 3–4 mice per experiment; ns, not significant. Statistical analyses were performed with ANOVA for multiple comparisons with Holm-Sidak’s correction. Error bars represent SEM. (TIF) [file ppat.1007583.s012.tif]
